# One Step Closer to Coatings Applications Utilizing Self-Stratification: Effect of Rheology Modifiers

**DOI:** 10.1021/acsapm.3c01288

**Published:** 2023-07-31

**Authors:** Timothy J. Murdoch, Baptiste Quienne, Maialen Argaiz, Radmila Tomovska, Edgar Espinosa, Franck D’Agosto, Muriel Lansalot, Julien Pinaud, Sylvain Caillol, Ignacio Martín-Fabiani

**Affiliations:** † Department of Materials, Loughborough University, LE11 1RJ Loughborough, United Kingdom; ‡ CNRS, ENSCM, ICGM, Univ Montpellier, 34293 Cedex 5 Montpellier, France; § POLYMAT and Departmento de Química Aplicada, Facultad de Ciencias Químicas, University of the Basque Country, UPV/EHU, Joxe Mari Korta Zentroa, Tolosa Hiribidea 72, Donostia-San Sebastian 20018, Spain; ∥ CPE Lyon, CNRS, UMR 5128, Catalysis, Polymerization, Processes and Materials (CP2M), Univ Lyon, Université Claude Bernard Lyon 1, 43 Bd du 11 novembre 1918, 69616 Villeurbanne, France

**Keywords:** colloids, stratification, self-assembly, polymers, rheology modifiers

## Abstract

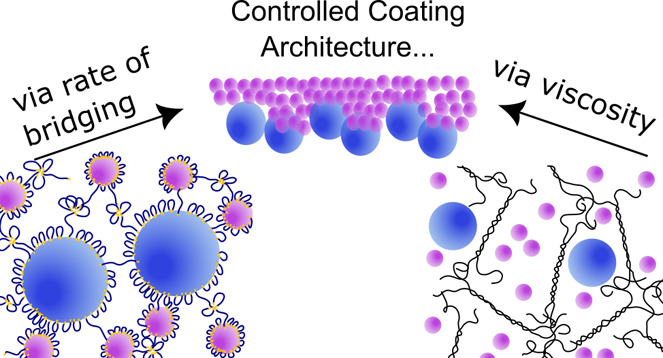

Self-stratification of model blends of colloidal spheres has recently been demonstrated as a method to form multifunctional coatings in a single pass. However, practical coating formulations are complex fluids with upward of 15 components. Here, we investigate the influence of three different rheology modifiers (RMs) on the stratification of a 10 wt % 7:3 w:w blend of 270 and 96 nm anionic latex particles that do not stratify without RM. However, addition of a high molar mass polysaccharide thickener, xanthan gum, raises the viscosity and corresponding Péclet number enough to achieve small-on-top stratification as demonstrated by atomic force microscopy (AFM) measurements. Importantly, this was possible due to minimal particle–rheology modifier interactions, as demonstrated by the bulk rheology. In contrast, Carbopol 940, a microgel-based RM, was unable to achieve small-on-top stratification despite a comparable increase in viscosity. Instead, pH-dependent interactions with latex particles lead to either laterally segregated structures at pH 3 or a surface enrichment of large particles at pH 8. Strong RM–particle interactions are also observed when the triblock associative RM HEUR10kC12 is used. Here, small-on-top, large-enhanced, and randomly mixed structures were observed at respectively 0.01, 0.1, and 1 wt % HEUR10kC12. Combining rheology, dynamic light scattering, and AFM results allows the mechanisms behind the nonmonotonic stratification in the presence of associative RMs to be elucidated. Our results highlight that stratification can be predicted and controlled for RMs with weak particle interactions, while a strong RM–particle interaction may afford a wider range of stratified structures. This takes a step toward successfully harnessing stratification in coatings formulations.

## Introduction

Drying multicomponent colloidal dispersions plays an essential role in a wide range of applications including decorative and protective coatings,^1^ photonic glasses,^2^ and electrode fabrication.^3^ In most cases, the distribution of components through the film is crucial to the film’s overall functionality. For example, a functional layer of nanoparticles at the top of a latex film can increase hardness,^4,5^ inhibit bacteria growth,^6^ or reduce tackiness,^7^ while retaining good adhesion to the substrate. However, controlling the distribution often requires multiple deposition steps. An alternate approach is self-stratification, where the assembly of components during drying allows formation of multilayer films in a single pass.^3,8,9^

Self-stratification of binary blends of colloidal spheres has been demonstrated for a wide range of systems.^8^ Among other factors, the distribution of particles is found to be highly dependent on the rate of diffusion (*D*), the evaporation rate (*E*), and the height (*H*) of the film, leading to characteristic diffusion (τ_diff_ ∼ *H*^2^/*D*) and evaporation times (τ_evap_ ∼ *H*/*E*). The ratio of these times is known as the Péclet number , which determines whether evaporation or diffusion is dominant. Particle accumulation at the evaporation front occurs if Pe > 1. In a stable binary blend of large (L) and small (S) particles, small-on-top stratification can occur for Pe_L_ > Pe_S_ > 1.

Several theories have been developed to predict the conditions for small-on-top stratification.^10−14^ Most of these models feature a gradient of small particles acting on the large particles, which causes a diffusiophoretic drift velocity of large particles relative to small particles. In general, larger size ratios of large:small particles (α), number ratios of small:large particles (*N*), and Pe_S_ favor stratification. One of the most adopted theories is the jamming front model of Sear.^10^ Here, stratification occurs only if there is sufficient time for the small particles to form a jammed layer at the evaporating interface and if the diffusiophoretic drift velocity of the large particles is large enough to outrun the jamming front.

In practice, coating formulations are not simple binary blends of hard spheres. For example, along with the binder and pigment, a realistic paint formulation contains around 10–15 different additives such as defoamers, wetting agents, and dispersants.^1^ These components can significantly alter particle–particle interactions, which in turn can significantly alter the degree and type of stratification.^9,15−17^ To bring the self-stratification method closer to paint and coating applications, the influence of these additives on the assembly process needs to be ascertained.

Polymeric rheology modifiers and thickeners are of particular importance in waterborne paints to achieve the desired relationship between viscosity and shear rate for each stage of film formation.^18^ However, studies of their effect on stratification are limited in number and scope.^19,20^ For example, a commercial alkali-swellable emulsion thickener was utilized to control Pe over several orders of magnitude.^19^ Stratification was observed only at intermediate Pe_S_, with an extension of Sear’s model to account for the decreased width of the accumulation zone used to explain the lack of stratification at very high Pe_S_. A commercial associative rheology modifier has also been used to control the diffusion and subsequent stratification of small molecules in a latex film.^20^ This allowed the degree of cross-linking to be varied through the depth of the film. However, these studies did not consider the influence of rheology modifier–particle interactions on stratification. Furthermore, the studies were conducted at α > 5, which favors small-on-top stratification, while we will demonstrate small-on-top stratification with α ∼ 3.

**Scheme 1:**
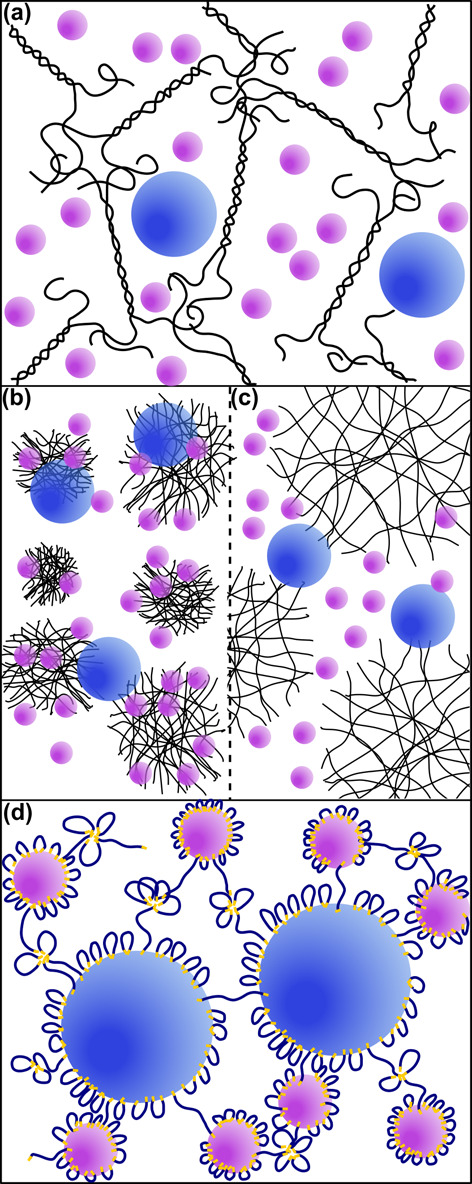
Structure of Latex Blend of Large (Blue) and Small (Purple) Particles in a (a) Nonassociative Xanthan Gum Solution, (b) Strongly Interacting, Collapsed Carbopol Microgel, (c) Weakly Interacting Swollen Carbopol Microgel, or (d) an Associative HEUR Rheology Modifier-Containing Dispersion

In this work, we will focus on the three rheology modifiers shown in Scheme 1. Xanthan gum (Scheme 1a) is a high molar mass (*M*_r_) linear polysaccharide that forms a network consisting of stiff hydrogen-bonded segments and entangled regions.^21,22^ This network and the corresponding rheology are found to be minimally affected by the addition of latex. Carbopol is a class of lightly cross-linked poly(acrylic acid) microgels that can swell up to 1000 times their dry size in basic conditions.^23^ pH-dependent interactions between particles and microgel have a significant impact on the dispersion rheology. Hydrophobically modified ethoxylated urethanes (HEUR) are a class of associative rheology modifier consisting of a hydrophilic poly(ethylene oxide) backbone end-capped with a hydrophobic alkyphenyl, fluorocarbon, or aliphatic alkyl chain.^24,25^ Note that for specific systems we will use HEURXXkCYY, where XXk is the *M*_r_ (g/mol) of the hydrophilic backbone and YY is the number of methylene units in the hydrophobic alkyl end groups. While often envisaged as a network of flowerlike micelles, in paints HEUR chains largely reside at the surface of latex particles.^26^ Therefore, the favorable shear thinning behavior of HEUR–latex dispersions results from the disruption of a network of colloids bridged by HEUR chains.

Herein, we compare the choice of rheology modifier to the stratification of a model coating formulation. This system consists of rheology modifier blended with a 7:3 binary mixture of latex particles where Pe_S_ > 1. In the absence of the rheology modifier Pe_S_, the size ratio of ∼3 and the volume fraction of small particles are too small to achieve small-on-top stratification. However, we show that a wide variety of stratified structures can be achieved depending on the balance of viscosity increase and the strength of rheology modifier–latex particle interactions. This work provides valuable insights into the interactions between key ingredients of paints, coatings, and other waterborne formulations and advances the current pathway of innovation in the self-stratification in colloidal blends.

## Materials and Methods

### Materials

Sodium hydroxide (reagent grade, anhydrous, ≥98%), hydrochloric acid (ACS reagent, ≥ 37%), and 24 × 24 mm^2^ glass coverslips were purchased from Fisher Scientific (Rugby, United Kingdom). All aqueous solutions were made with ultrapure water (18.2 MΩ cm^–1^, Suez Select Fusion). Poly(ethylene glycol) (10000 g mol^–1^) was purchased from Alfa Aesar (Kandel, Germany). Dibutyltin dilaurate (DBTDL), dodecyl isocyanate, toluene, and diethyl ether were purchased from Sigma-Aldrich Merck (Darmstadt, Germany). The NMR solvent CDCl_3_ was purchased from Eurisotop (Saint-Aubin, France). All materials were used as received. Xanthan gum from *Xanthomonas campestris* was purchased from Sigma-Merck and used as received. Carbopol 940 was purchased from Acros Organics and used as received. For simplicity we refer to this as Carbopol throughout.

Large latex particles were produced via surfactant-free seeded emulsion polymerization. The particles comprised poly(butyl acrylate-*co*-methyl methacrylate) (P(BA-*co*-MMA), MMA:BA, 1:1 by weight) and were obtained in the presence of 1 wbm % sodium styrenesulfonate (NaSS) yielding an overall solids content of 50 wt % according to a protocol provided elsewhere.^27^ The resulting particles had a diameter at an intensity peak of 269 nm, a polydispersity index (PDI) of 0.02, and a zeta potential of −46.5 mV, as determined by dynamic and electrophoretic light scattering. Small latex particles with comparable P(BA-*co*-MMA) cores were synthesized by polymerization-induced self-assembly (PISA). Briefly, a poly(sodium styrenesulfonate) (PNaSS) macromolecular reversible addition–fragmentation chain transfer (macroRAFT) agent (number-average molar mass (*M*_n_) 2700 g mol^–1^) was synthesized by RAFT in the presence of 4-cyano-4-thiothiopropylsuflanylpentanoic acid (CTPPA). Chain extension in the presence of BA and MMA (1:1 by weight) resulted in latex particles stabilized by 19.5 wt % of PNaSS with an overall solids content of 20.9 wt %. These particles had a diameter, PDI, and zeta potential of 102 nm, 0.02, and −56.7 mV, respectively. Both large and small particle dispersions had a native pH of 5.5 when diluted to 10 wt % in deionized water. Detailed procedures for the macroRAFT agent and PISA are provided elsewhere.^28,29^

HEUR10kC12 was synthesized as per earlier work,^25^ except that the molar mass of poly(ethylene glycol) was 10000 g mol^–1^ and dodecyl isocyanate was used to create HEUR10kC12. Here, 7.5 g (1 equiv) of poly(ethylene glycol) (10000 g mol^–1^) and 0.016 g of DBTDL (0.2 wt % of the total mass) were solubilized in 15 mL of dried toluene. After azeotropic distillation and removal of the residual water traces, 0.333 g of dodecyl isocyanate (1.05 equiv) was added to the mixture at 80 °C under stirring and an argon atmosphere. After 2 h of reaction, 10 mL of toluene was added, and the heating was stopped. Once the solution was back to room temperature, the polymer was precipitated in 300 mL of diethyl ether to remove the remaining isocyanates, urea traces, and catalyst. The polymer obtained, HEUR10kC12, was then washed with diethyl ether before drying under vacuum. A white powder was finally obtained with a yield of 89%. ^1^H NMR (400 MHz, CDCl_3_): δ 0.86 (t, CH_3_), 1.24 (s, CH_2_), 1.47 (t, CH_2_), 3.14 (q, CH_2_), 3.63 (s, CH_2_), 4.19 (t, CH_2_).

### Latex Film Preparation

HEUR containing dispersions were prepared from stock latex solutions diluted to 10 wt % solids with deionized (DI) water. These solutions were added to a preweighed mass of HEUR to form dispersions with up to 1 wt % HEUR. The dispersions were vortex mixed for 15 s and then pH adjusted with HCl and NaOH solutions. These dispersions were stirred for at least 16 h, after which the pH was checked and readjusted, if necessary, prior to casting. Xanthan gum and Carbopol dispersions were formed in a similar manner with the exception of the lowest concentration of xanthan gum. Here, 0.708 g of the 50 wt % large particle stock, 0.726 g of the 20.9 wt % small particle stock, 0.010 g of 2 wt % xanthan gum gel, and 3.614 g of DI water were combined to create a 10 wt % 7:3 (w/w) large:small dispersion with 0.004 wt % xanthan gum prior to mixing.

Prior to casting, glass coverslips (24 × 24 mm^2^) were cleaned in an UV-ozone cleaner (Ossila, Sheffield) for 10 min. 200 μL of each dispersion was then drop-cast onto a glass coverslip. Note that substrate chemistry is not expected to affect stratification of the latex dispersions.^30^ In the case of HEUR dispersions, additional films were formed by diluting by the existing dispersion by a factor of 100 in DI water. The diluted dispersion was then cast with a volume of 20 μL. Dispersions were dried in ambient laboratory conditions at 21 ± 1 °C and a relative humidity ∼40% over a period of 3–4 h, yielding a final film thickness of approximately 30 μm.

### Shear Rheology

Steady shear rate sweeps were performed by using a TA Instruments DHR-2 controlled stress rheometer using a 40 mm diameter stainless steel parallel plate geometry. The temperature was maintained at 25 °C by using a Peltier plate. In most cases, a micropipet was used to apply 535 μL of sample directly to the bottom plate. This volume was calibrated at the measurement gap of 0.4 mm by systematically varying the volume of DI water until surface tension artifacts were minimized.^31^ No additional preshear or flow-conditioning was performed prior to measuring. Data were collected between 0.1 and 1000 s^–1^ with between 3 and 5 logarithmically spaced points per decade. Even with the volume correction, useful data were only measurable above 10 s^–1^ for solutions with viscosity close to water. A spatula was used to load and trim high viscosity samples.

### Dynamic Light Scattering (DLS) and Zeta Potential

DLS and zeta potential measurements were performed by using a Malvern Zetasizer Ultra (Malvern Panalytical, Malvern). The 10 wt % latexes were diluted to 0.05 wt % in either DI water or the target rheology modifier solution for both DLS and zeta potential measurements. HCl and NaOH solutions were used to adjust the pH of the diluted solutions, followed by at least 1 h of equilibration prior to measurement.

Backscatter DLS data were collected at 25 °C with a minimum of 3 runs for each sample. The zeta potential measurements were performed in Malvern DTS1070 folded capillary cells. Data were analyzed using the default general-purpose algorithms in the Malvern ZS XPLORER software. Polydispersity index values between 0.01 and 0.25 were found, depending on the rheology modifier concentration. This led to a large variation in the ratio between *Z*-average diameter and intensity distribution peak diameter. Therefore, the intensity peak diameter was chosen for presentation.

### Atomic Force Microscopy (AFM)

AFM topography measurements were performed with a Bruker BioScope Resolve (Bruker, Santa Barbara, CA). The majority of measurements were performed in PeakForce QNM mode with silicon nitride probes (ScanAyst air) with nominal tip radii and spring constants of 2 nm and 0.4 N m^–1^, respectively. The 5 × 5 μm^2^ scans were taken at a minimum of three different locations on the sample. Measurements on films formed after dilution were conducted using tapping mode. These measurements were performed with silicon probes (RTESPA300) with nominal resonant frequency, tip radii, and spring constant of 300 kHz, 8 nm, and 40 N m^–1^, respectively. Images were analyzed with using the open source Gwyddion 2.61 software.^32^ Here, images were corrected by subtracting a second-order polynomial background. The number distribution of each particle size was extracted by using the built-in watershed procedure, which allowed calculation of the number ratio. This procedure was greatly improved by first applying a local contrast filter with a 0.02 μm kernel size to increase the contrast between the edges of the particles.

## Results and Discussion

### Rheology Modifier-Free Films

**Figure 1:**
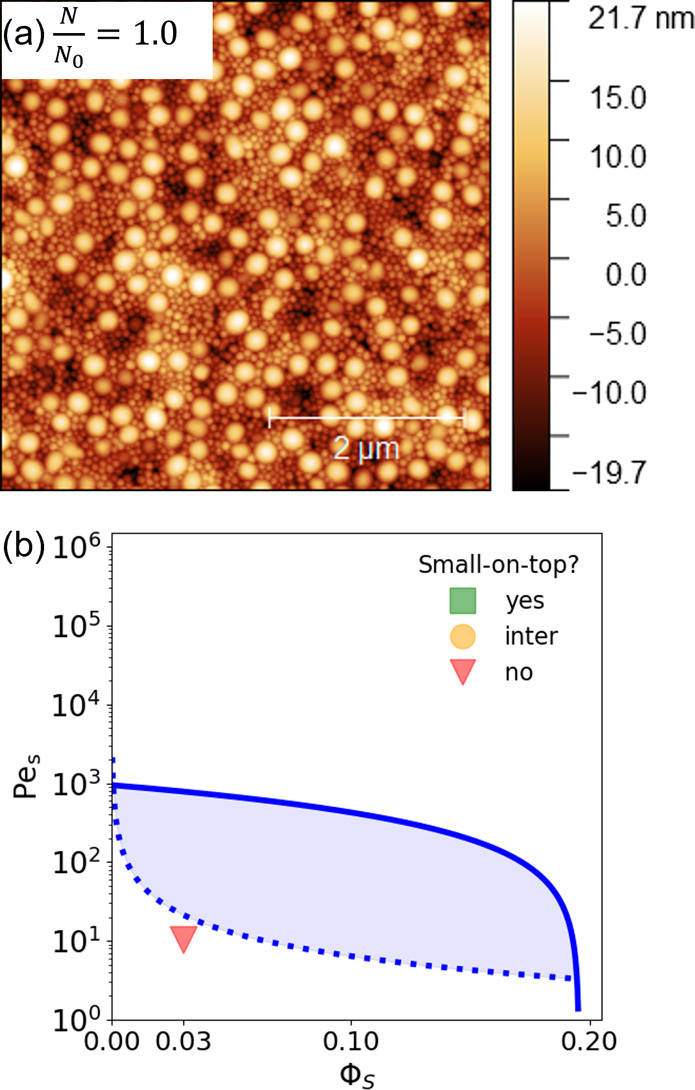
(a) 5 × 5 μm^2^ AFM topography image of rheology modifier-free binary latex film. (b) Pe_S_ and ϕ_S_ parameter map for stratification of binary colloid mixtures. The shaded region corresponds to regions where small-on-top stratification is predicted.

Coating formulations are multicomponent mixtures where species interact in a complex way. However, the interplay between components can be understood by building up the complexity through model systems. Our model system consists of a 7:3 (w/w) blend of large and small latex particles with an overall particle concentration of 10 wt %. The topography of the neat latex film is shown through the atomic force microscopy (AFM) measurements in Figure 1a. Image analysis in Gwyddion shows that the number ratio (*N*) of large:small particles at the top surface matches that of the bulk solution (*N*_0_ ∼ 1:9.5), i.e., *N*/*N*_0_ = 1. While 1 < Pe_S_ < Pe_L_, the homogeneous distribution is still consistent with the extended jamming front model^19^ as the location of Pe_s_ and ϕ_s_ on the parameter map for stratification of binary colloid mixtures in Figure 1b falls below the shaded region where small-on-top stratification is predicted.

### Dispersion Rheology

In laboratory conditions, the effect of the Péclet number on stratification is often controlled by changing the temperature and humidity. However, these parameters are impractical in many real-world applications. An alternate approach is to utilize rheology modifiers to control the formulation viscosity. Several classes of rheology modifiers are used in waterborne paints and inks. In this study, we compare the effect of a high-*M*_r_, linear polyelectrolyte (xanthan gum), a microgel-based thickener (Carbopol), and an associative triblock copolymer (HEUR10kC12) on the stratification of binary latex blends. However, the rheology of dispersions needs to be understood before their influence on stratification can be elucidated. The compositions of these dispersions are informed by typical thickener concentrations in paints such as 0.1–0.5 wt % for xanthan gum^33^ or 0.5–1.5 wt % for HEUR.^18^

**Figure 2:**
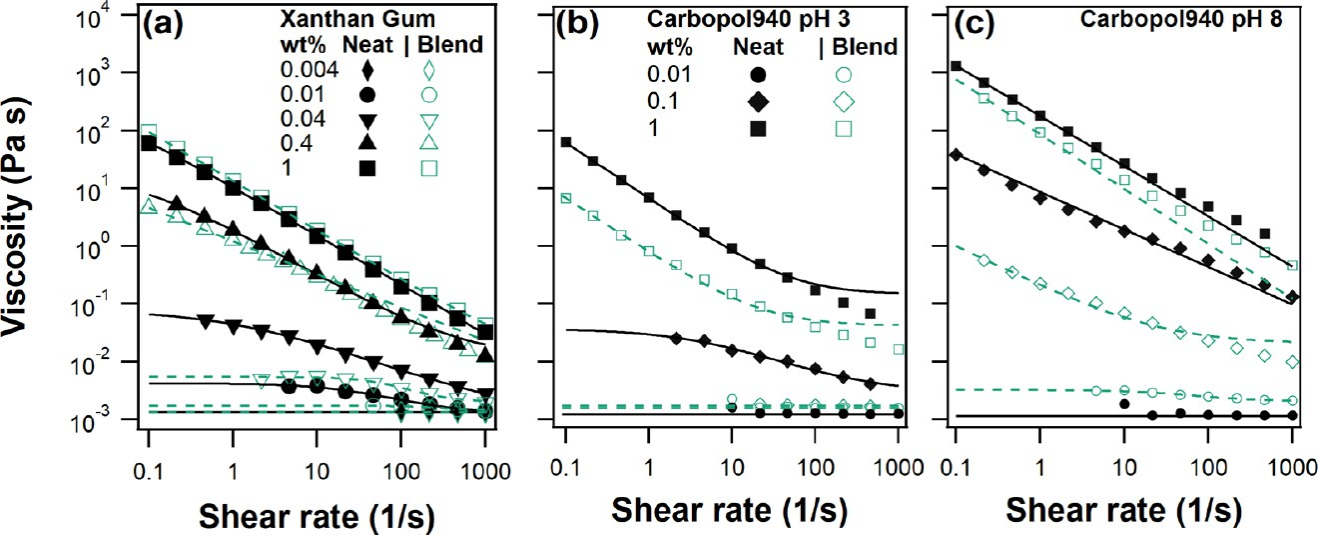
Steady shear viscosity vs shear rate for dispersions containing (a) as-prepared xanthan gum (pH 5.5), (b) Carbopol 940 at pH 3, and (c) Carbopol 940 at pH 8. All lines correspond to fits to eq 1 with the exception of 1 wt % Carbopol 940 (pH 8), which is fit to a power law. Solid lines and symbols correspond to latex free solutions, while dotted lines and empty symbols correspond to solutions with a 10 wt % 7:3 large/small blend.

The steady shear rheology of neat xanthan gum solutions is Newtonian at the lowest studied concentration of 0.004 wt % as shown in Figure 2a. As the concentration is increased, a Newtonian plateau is observed, followed by a shear thinning region. At concentrations of 0.4 wt % and higher, only shear thinning is present. In all cases, the shear thinning region is well described by a power law of the form η ∝ γ̇^*n*^, where η is the viscosity, γ̇ is the shear rate, and *n* is the power law index. Furthermore, the full range of η vs γ̇ is well described by the Cross model:^21^
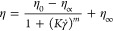
1where η_0_ is the zero-shear rate viscosity, η_∞_ is the infinite shear rate viscosity, *K* is the characteristic time of the solution, and *m* is the rate index.

Dispersions containing the 10 wt % small/large latex blend in addition to xanthan gum exhibit almost identical rheology to the corresponding latex-free solutions at 0.4 and 1 wt %. At 0.04 wt % and below, there is a minor reduction in the viscosity of the blend versus relative to latex-free solutions. This coincides with the entanglement concentration of xantham gum.^21^ However, overall, the rheology of the blends indicates a negligible influence of latex on the xanthan gum network in pH 5.5 solution. This is a result of the strong double-layer repulsion between the anionic latex and negatively charged xanthan gum (p*K*_a_ ∼ 4).^34^

The rheology of latex-free Carbopol is shown in Figure 2b,c. At pH 3 the concentration-dependent viscosity and degree of shear thinning are comparable to those of equivalent xanthan gum solutions. Therefore, eq 1 is used to fit the data. Here, the pH is below the reported p*K*_a_ of Carbopol of 6.0 ± 0.5,^35^ meaning that the carboxylic acid groups should be significantly protonated (i.e., neutralized). Increasing the pH to 8 results in a significant increase in viscosity, as the microgel particles swell due to electrostatic repulsion between the deprotonated carboxylic acid groups. Here, at 1 wt % fits to eq 1 are unstable as the power law region spans the shear rate range.

Combining the 10 wt % latex dispersion with Carbopol results in a significant decrease in viscosity at a given shear rate (Figure 2b,c). This decrease in viscosity suggests that there are significant particle–Carbopol interactions. Furthermore, for a given Carbopol concentration, the decrease in viscosity is larger at pH 3 than at pH 8, which suggests that the interaction involves the protonated carboxylic acid moiety. The most likely candidates for these interactions are nonelectrostatic hydrogen-bonding, dipole–ion, and dipole–dipole interactions between the protonated carboxylic acid moieties of Carbopol and the sulfonate moieties of the latex particles. Similar interactions are believed to drive adsorption of poly(acrylic acid) onto comparable sulfate stabilized latex particles.^36^

In contrast to the other rheology modifiers in this study, both latex-particle-free and latex-particle-containing HEUR10kC12 dispersions do not display a large increase in viscosity relative to water and are predominantly Newtonian (Figure S1). While HEUR solutions are often envisaged as shear-thinning, for relatively short C12 alkyl moieties the corresponding low relaxation time can result in largely Newtonian behavior at concentrations as high as 5 wt %.^18,37^ The rheology of these systems is summarized in Figure S2 by plotting the viscosity relative to water as a function of the HEUR10kC12 concentration. Here, any non-Newtonian behavior is captured by plotting values at shear rates of 1 and 1000 s^–1^. The viscosity of the latex-free HEUR10kC12 solutions remains close to water up to 0.2 wt %, above which there is an exponential increase in viscosity with increasing concentration. This increase likely corresponds to the transition from flowerlike micelles to a network structure.

Similar to the neat HEUR10kC12 solutions, the viscosity of the latex–HEUR10kC12 systems does not vary significantly for rheology modifier concentrations up to 0.1 wt %. However, at 1 wt % the rheology depends on the particle identity. Here, large particles–HEUR10kC12 dispersions have a lower viscosity than expected from their individual viscosity. This subadditive behavior is consistent with a colloid-centered system, where the majority of the HEUR10kC12 chains are associated with the latex particle.^18^ Furthermore, the increased viscosity relative to the neat large particle dispersion can then be interpreted as an increase in the effective colloid volume fraction.

In contrast to the large particles, dispersions containing small particles display irreversible shear thinning behavior at 1 wt % HEUR10kC12 due to the presence of aggregates that are not present at lower HEUR10kC12 concentrations (Figure S1). These solutions settle over a period of hours but are easily redispersed by gentle shaking or stirring. The polymer-induced aggregation does not appear to depend on the differing concentration of styrenesulfonate groups at the surface of the latex as we will later show that the degree of HEUR10kC12 adsorption is similar for both particles in the colloid-centered regime. Instead, the most likely explanation for the aggregation is lower nominal interparticle distance. The interparticle distance is proportional to the particle radius, with values at a volume fraction of 0.1 of ∼80 and ∼230 nm expected for small and large particles, respectively (see Table S1). Furthermore, qualitative tests show that 30 wt % large particle dispersions with an interparticle distance around 80 nm leads to aggregation in 3 wt % HEUR10kC12, while 2 wt % small particles in 1 wt % HEUR10kC12 with an interparticle distance of ∼210 nm are stable. Smaller interparticle distances promote polymer bridging and can lead to stronger shear thinning behavior for smaller particles.^38,39^

### Effect of Nonassociative Rheology Modifiers on Stratification

**Figure 3:**
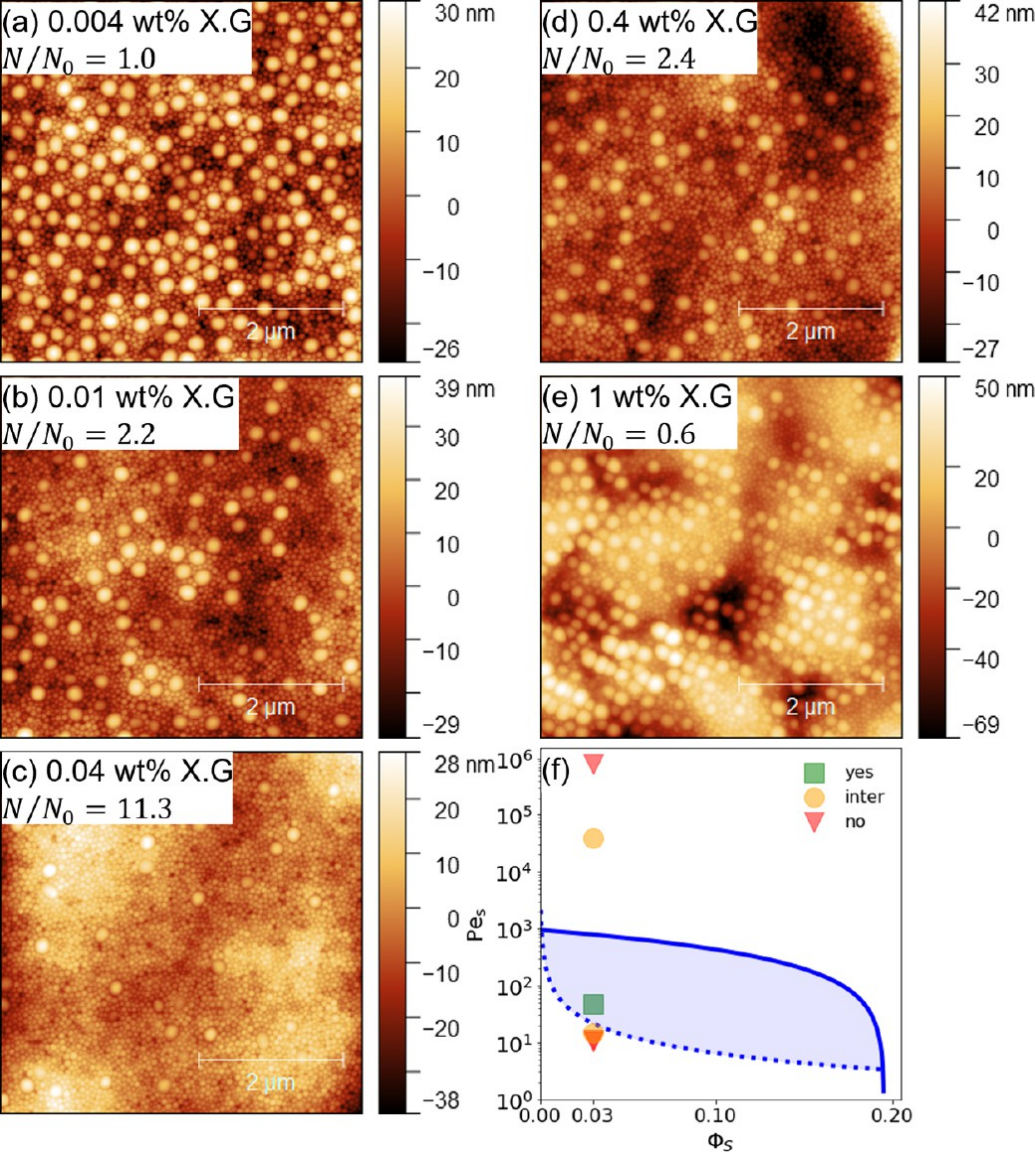
(a–e) 5 × 5 μm^2^ AFM topography image of binary latex films with increasing concentrations of xanthan gum. (f) Pe_S_ and ϕ_s_ parameter map for stratification of binary colloidal mixtures. The shaded region corresponds to regions where small-on-top stratification is predicted. Note that Pe_S_ increases monotonically with the xanthan gum concentration.

Adding up to 1 wt % xanthan gum to 10 wt % latex solutions can increase the low shear viscosity and corresponding Péclet number by over 4 orders of magnitude. AFM measurements in Figure 3 demonstrate the effect of this increasing Péclet number on the composition of the top layer of the film. Between 0.004 and 0.04 wt % xanthan gum, the Pe_S_ increases from 11 to 47 along with a trend from a homogeneous distribution to small-on-top stratification. Further increasing the concentration to 0.4 and 1 wt % increases the Pe_S_ up to 80 × 10^5^. Here, the trend reverses with a minor enhancement in small and then large particles on top, as Pe_S_ is increased. Furthermore, at the highest xanthan gum concentration there is an increase in micrometer scale roughness of the surface.

The effects of xanthan gum on latex stratification are compared to the extended jamming front model in Figure 3f.^19^ Here, the shaded area corresponds to the region where small-on-top stratification is predicted. In general, there is good agreement between experiment and theory with the only exception occurring at 0.4 wt % where intermediate, rather than the predicted no, stratification occurs. However, this is an encouraging result as the model is formulated in the limit of large size ratio and does not include the possibility of the xanthan gum stratifying or deviations from hard-sphere interactions. Similar results were achieved in the literature where Pe_S_ was varied by using temperature, film thickness, and two concentrations of alkali swellable emulsion (ASE) thickener comparable to Carbopol, whereas we test the model with polymer concentration as the only variable.^19^ Furthermore, ASE–particle interactions are not tested, while rheology measurements in Figure 2a suggest minimal rheology modifier–particle interactions. Altogether, these results highlight the ability of nonadsorbing rheology modifiers to control diffusiophoresis-based stratification even at a relatively low size ratio compared to typical studies of latex size segregation in the absence of any other additives.^8^

### A Comparison of Strongly and Weakly Interacting Microgel Rheology Modifiers on Stratification

Changes in the degree of latex–Carbopol interaction with pH lead to large changes in the Péclet number of the small particles and therefore in the final film architecture. For example, at 0.01 wt % Pe_S_ is 13 at pH 3 and 28 at pH 8. Here, the jamming front model would predict no stratification and small-on-top stratification, respectively. However, neither of the predicted structures occurs, suggesting a deviation from pure diffusiophoresis driven stratification. Instead, both pH values show a minor enhancement in the number of large particles in the top of the film relative to the bulk suspension with *N*/*N*_0_ between 0.6 and 0.7 (Figure S3).

**Figure 4:**
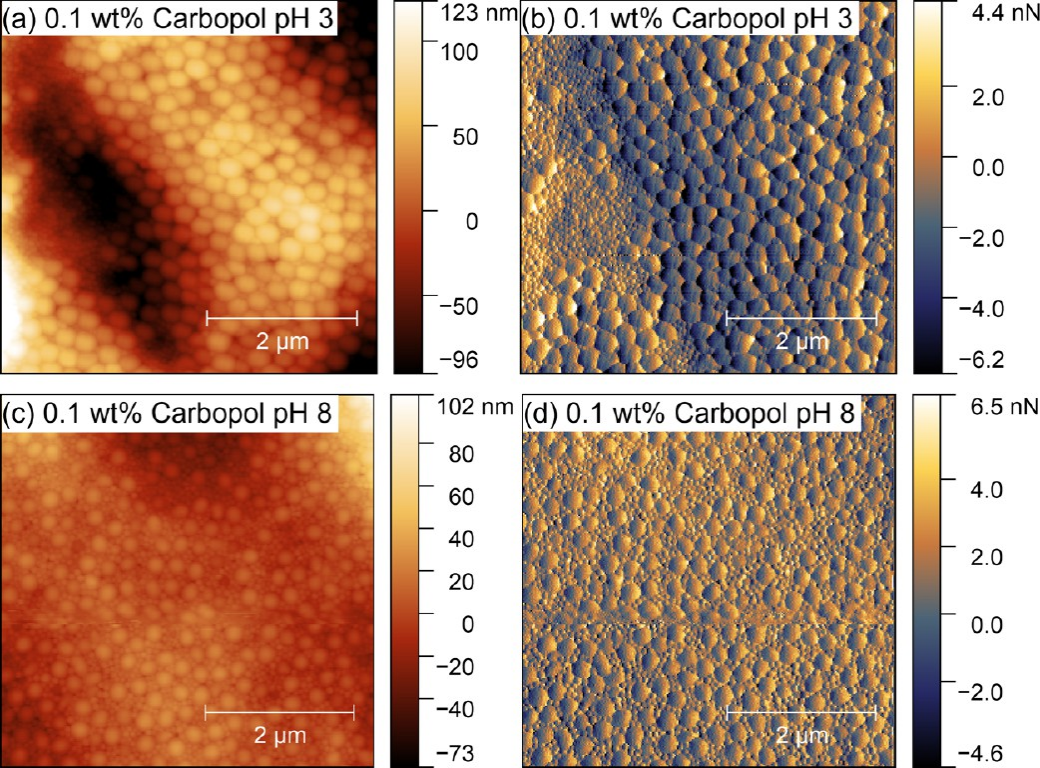
5 ×5 μm^2^ AFM topography images of films formed from 10 wt % latex blend with 0.1 wt % Carbopol at (a) pH 3 and (c) pH 8. Corresponding maps of adhesion are provided in (b) and (d) for pH 3 and 8, respectively. Adhesion is presented in arbitrary units on the order of 1 nN.

In contrast to 0.01 wt % Carbopol, the structures formed from dispersions containing 0.1 and 1 wt % Carbopol are strongly dependent on the pH rather than the Carbopol concentration. Therefore, 0.1 wt % is chosen to present the pH-dependent structures in Figure 4. Here, the relatively large overall variation in the surface height obscures fine details of the particle distribution. Therefore, adhesion maps are provided in Figure 4 to enhance the local contrast. At pH 3, lateral segregation of the particle size is observed at both of the higher Carbopol concentrations. Again, this structure is not predicted by diffusiophoresis-based theories of stratification. In contrast, at pH 8 the structure is not significantly changed from 0.01 wt % aside from the presence of some particle free regions observable at 1 wt % Carbopol (Figures S3 and S4). These particle-free regions likely correspond to bare Carbopol.

The rheology of Carbopol suggests weaker rheomodifier–latex interactions at pH 8 than at pH 3. Weaker interactions may partly account for the minimal change in topography with increasing Carbopol concentration at pH 8, despite Pe increasing by several orders of magnitude. More importantly, the microviscosity of the interstitial regions between Carbopol microgel particles has been found to be comparable to water.^40^ This suggests Carbopol and other similar microgel-based thickeners that do significantly affect the viscosity at the submicrometer level are unsuitable for controlling diffusiophoresis-based stratification.

The lateral segregation observed at pH 3 may arise from the preferential adsorption of particles onto patches of the Carbopol microgels. This is plausible due to different concentrations of sodium styrenesulfonate groups on the surface of the two latex particle populations. However, the relatively large overall size and high size dispersity of the Carbopol microgels mean that further elucidation of the mechanism is difficult.

### Nonmonotonic Relationship between Associative Rheology Modifier Concentration and Stratification

#### HEUR Concentration Effects on Stratification and Adsorption

The change in viscosity of the HEUR–latex dispersions in this study is small relative to that of the other rheology modifiers. In fact, according to the jamming front model,^19^ the only condition where the increase in viscosity and corresponding Pe_S_ is large enough to predict small-on-top stratification is at 1 wt % HEUR10kC12. Therefore, these dispersions elucidate the effect of rheology modifier–latex interactions on stratification in the absence of significant changes in Péclet number.

**Figure 5:**
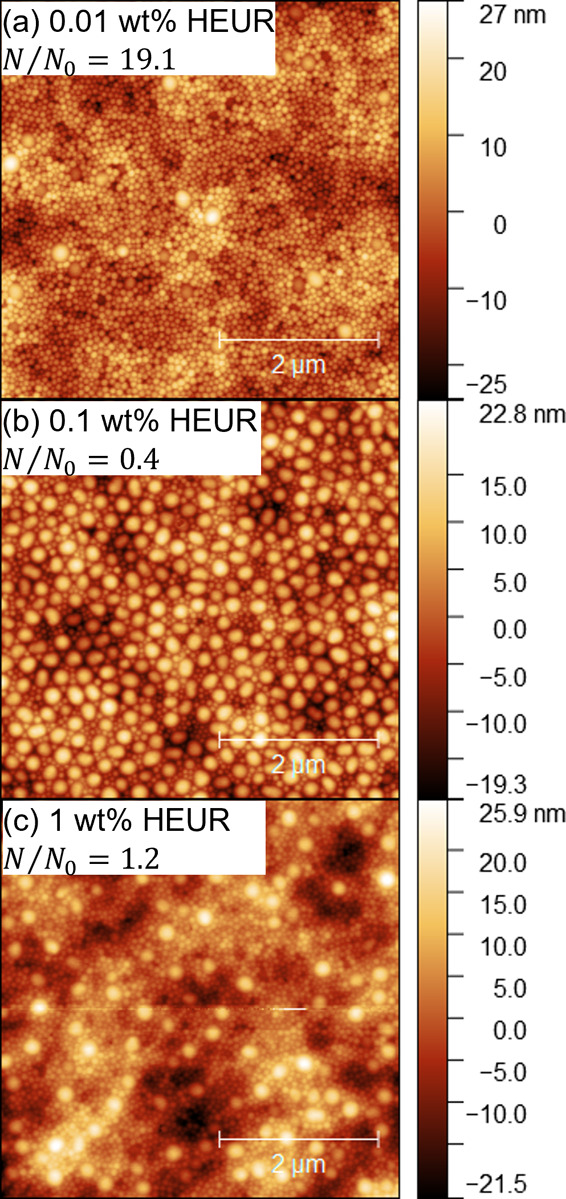
5 × 5 μm^2^ AFM topography images of films formed from 10 wt % latex blends with (a) 0.01, (b) 0.1, and (c) 1 wt % HEUR10kC12 at the unadjusted pH of 5.0–5.5.

AFM measurements in Figure 5 demonstrate a nonmonotonic relationship between film structure and HEUR concentrations between 0.01 and 1 wt %. At the lowest concentration of 0.01 wt %, small-on-top stratification occurs (*N*/*N*_0_ = 19.1). Increasing the concentration to 0.1 wt % results in an enhancement of large particles at the top of the dried film (*N*/*N*_0_ = 0.4). Qualitatively, there is a mixture of large particles separated by a single small particle and direct large–large contacts. Further increasing the concentration to 1 wt % leads to no stratification (*N*/*N*_0_ = 19.1). Note that similar structures were achieved in preliminary experiments with dispersions containing a volume fraction ratio of 85:15 large:small particles (Figure S5). This further shows that these structures must result from the HEUR–latex interactions.

**Figure 6:**
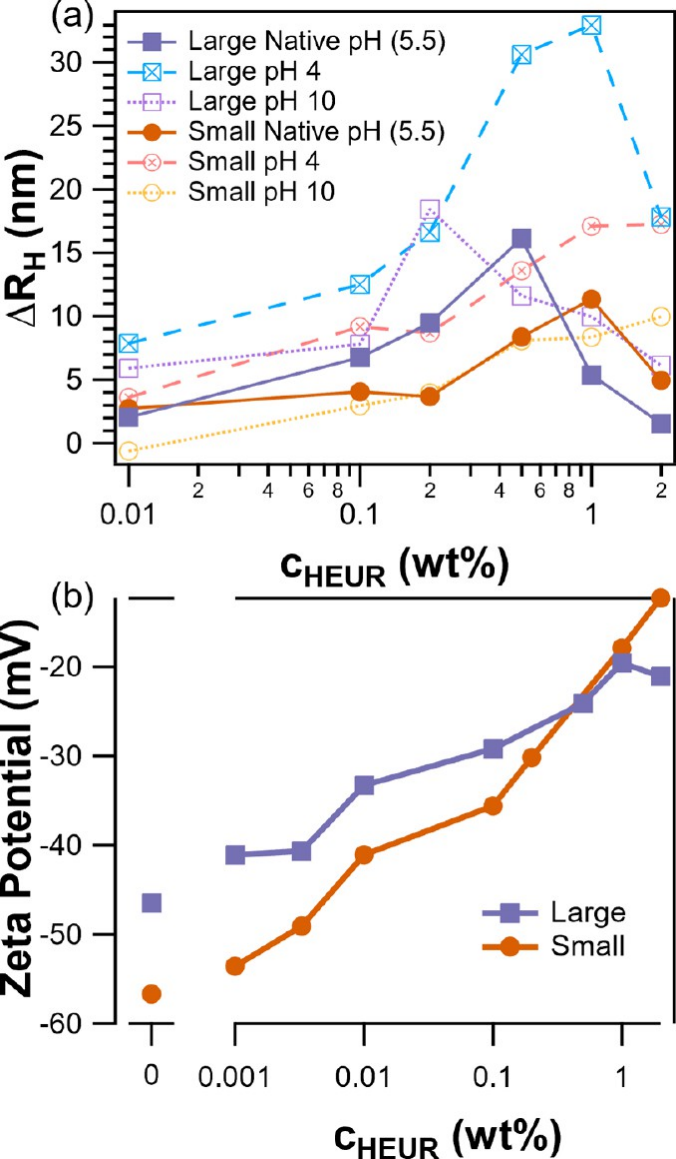
(a) Change in apparent hydrodynamic diameter of latex particles due to the adsorption of HEUR10kC12 measured by DLS. (b) Zeta potential as a function of the HEUR10kC12 concentration.

Insight into interactions between latex and HEUR in the limit of excess HEUR is possible through the DLS measurements summarized in Figure 6a. Intensity versus size distributions are provided in Figures S6 and S7. These DLS measurements capture the change in the apparent hydrodynamic radius of the particle due to the formation of a layer of adsorbed HEUR10kC12. At the unadjusted pH of 5.0–5.5, both particle types have an adsorbed layer on the order of 2 to 3 nm at 0.01 wt % HEUR10kC12. This value is comparable to the radius of gyration of an equivalent poly(ethylene glycol) chain^41^ and is typical of thickness of polymers end-attached at low areal densities.^42^ Increasing the HEUR concentration to 0.1 wt % leads to an increase in the adsorbed layer thickness to around 6 nm for the large particles and 3 nm for the small particles. The increase in thickness suggests an extended, brushlike conformation (see the Supporting Information).^43^

The apparent adsorption of HEUR onto the latex particles is sensitive to particle type at concentrations above 0.1 wt % HEUR. In the case of large particles, the adsorbed layer thickness increases to a maximum value of 16 nm at 0.5 wt %, followed by a sharp decrease with further increase in concentration. In contrast, small particles reach a maximum value of 10 nm at 1 wt % HEUR, followed by a reduction in thickness at 2 wt %. In both cases, the large maximum layer thicknesses can be accounted for by the formation of weakly associated layers of admicelles. These weakly associated layers are consistent with experiments^43,44^ and simulations^45^ of telechelic polymers.

Nonmonotonic adsorption of HEUR8kC12 on latex particles has been observed using centrifugation.^46^ For a range of surface acid concentrations, initial maxima occur at <0.1 wt % HEUR8kC12, followed by minima around 0.3 wt %, before a monotonic increase in adsorbed amount up to 0.6 wt %. The authors attributed the minima to bridging flocculation, reducing the accessible area for adsorption. Furthermore, the minimum adsorbed amount was inversely proportional to the number of charged species at the surface, as HEUR is thought to primarily interact through the hydrophobic end groups. This parallels our DLS data where the large particles containing ∼20 times fewer charge sites exhibit stronger desorption with increasing concentration than the small particles. However, even with a moderate increase in the polydispersity index (PDI) from ∼0.02 to ∼0.2 (Figure S8) with HEUR concentration, the change in thickness from our DLS data is much less than that required for dimer formation. Therefore, bridging flocculation is unlikely.

The concentration range where the apparent adsorbed layer thickness begins to decrease overlaps the concentration where network formation begins and the viscosity of latex-free HEUR solutions begins to increase. Given the low volume fraction of particles in the DLS experiments (ϕ ∼ 0.005), the HEUR network would not be expected to be significantly depleted, and the overall viscosity should be close to that of a latex-free HEUR10kC12 solution.^18^ Therefore, an alternate explanation for the apparent decrease in thickness is that the admicelles are incorporated into the HEUR network, with the apparent diffusion rate and apparent layer thickness of the particles measured by DLS corresponding only to HEUR strongly associated with the particle surface.

Another method for qualitatively looking at HEUR–latex adsorption is zeta potential measurements, as shown in Figure 6b. Adsorption of HEUR on latex causes the magnitude of the zeta potential to tend toward zero as the polymer layer shifts the electric double layer slipping plane away from the particle surface.^47^ This is largely the case in the current study, where both particles display a monotonic decrease in the magnitude of the zeta potential with increasing HEUR concentration. The largest difference in zeta potential between the two particle types occurs at concentrations below 0.01 wt %. These lower concentrations correspond to undersaturated surface coverages and are more representative of the colloid centered conditions found in the 10 wt % latex dispersions (see Table S2 and the surrounding discussion). Here, the absolute value of the zeta potential is smaller for the large particles. This may favor their presence at the top coating surface when competing against small particles with a stronger surface charge.^30^

#### Effect of pH on HEUR–Latex Interactions

**Figure 7:**
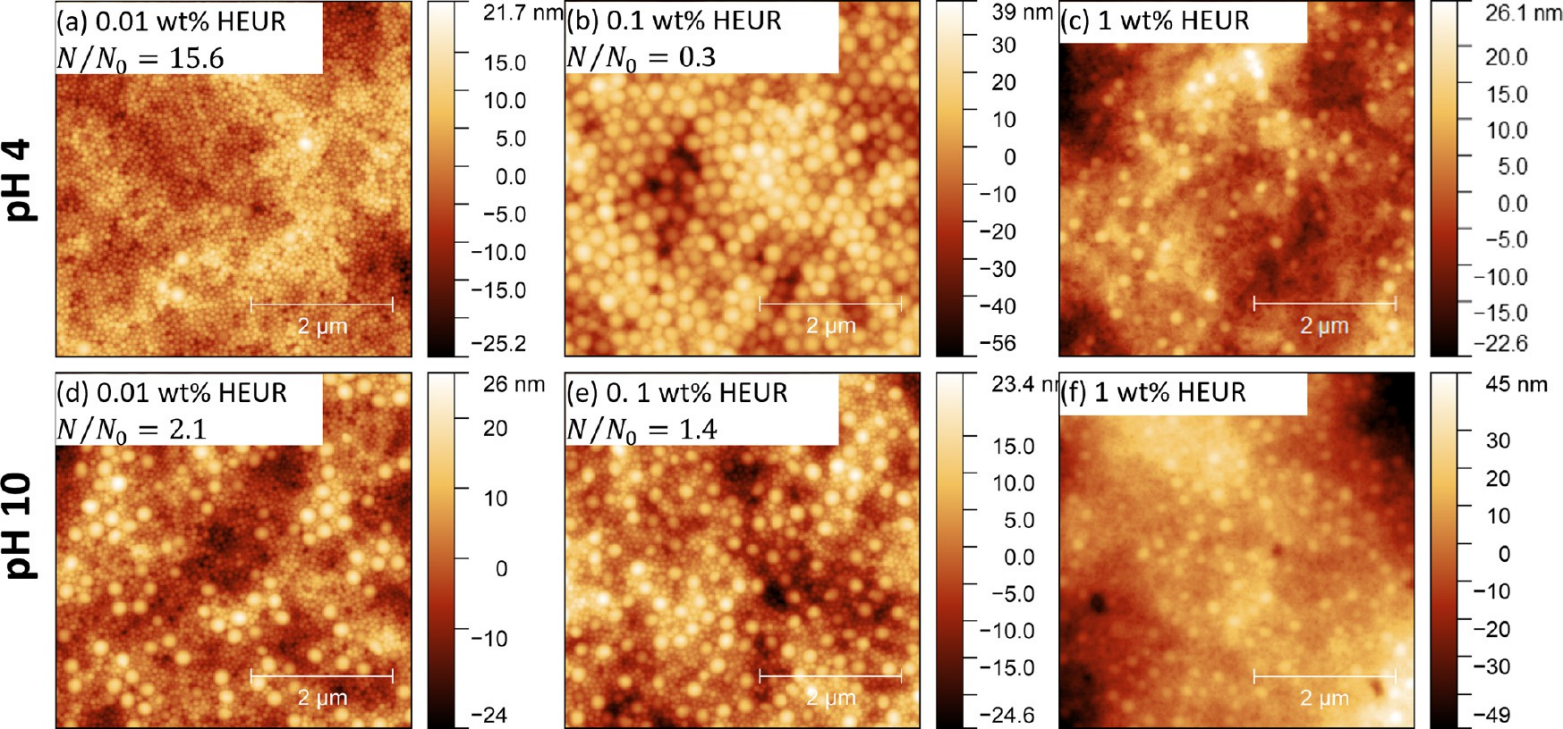
5 × 5 μm^2^ AFM topography images of films formed from 10 wt % latex blend with (a, d) 0.01, (b, e) 0.1, and (c, f) 1 wt % HEUR10kC12. Images in the top row (a, b, c) were cast at pH 4 while the bottom row (d, e, f) corresponds to films cast at pH 10.

Aside from the sodium styrenesulfonate content, a key difference between the large and small particles is the presence of a carboxylic acid moiety at the end of the PSSNa chains due to the use of CTPPA as the chain transfer agent during synthesis. Therefore, to investigate the role of these pH responsive groups, the topography of films cast at pH 4 and 10 is shown in Figure 7. These pH values were chosen to both span the p*K*_a_ of the COOH moieties of poly(acrylic acid)^48^ and to keep the ionic strength of acid and base conditions the same. Interestingly, the topography is found to be highly pH sensitive below 1 wt % HEUR10kC12, where we expect undersaturated adsorption. The structure at pH 4 is similar to those of equivalent unadjusted films. However, the films cast at pH = 10 show minimal stratification.

The topography at 1 wt % HEUR10kC12 appears to be independent of pH. However, *N*/*N*_0_ cannot be quantified due to the significant amount of HEUR present. Further insight into the effect of pH at this concentration is possible by mapping adhesion, as shown in Figure S9. Adhesion data at pH 10 show a monomodal distribution centered around 2.2 nN. The lighter regions in the corresponding inset image (Figure S9i) correspond to the interface between particles and are consistent with an increased tip–sample contact area at the bottom of local valleys.

In contrast to pH 10, adhesion data at pH 4 show a peak value around 2.6 nN and a smaller shoulder around 2.2 nN. The inset image (Figure S9ii) shows that the lower adhesion shoulder corresponds with large particles, while the higher adhesion is present everywhere else. Note that higher adhesion at pH 4 is also present to a lesser degree at 0.1 wt % HEUR. The origin of higher adhesion is likely due to a greater amount of HEUR present at the top interface at pH 4. Indeed, individual force curves show signatures of HEUR molecules being stretched (Figure S10). Importantly, this demonstrates that latex–HEUR interactions may affect the distribution of both latex and HEUR at the top of the film.

The effect of pH on HEUR–latex interactions is also apparent in the DLS data shown in Figure 6a. Note that at a given concentration of HEUR10kC12, adjusting the pH results in changes of the apparent thickness of the adsorbed HEUR. This may result from the increased ionic strength screening the sodium styrenesulfonate moieties and making hydrophobic adsorption more favorable. With small particles, the thickness values are nearly identical up to 0.5 wt % HEUR, with pH 4 resulting in slightly higher thicknesses at 1 and 2 wt %. In contrast, pH has a significant impact on large particles data with pH 4 displaying a larger maximum thickness of 32 nm, compared to 23 nm at pH 10. Furthermore, the maximum occurs at a HEUR concentration of 1 wt % at pH 4 compared to 0.05 wt % at the unadjusted condition and 0.2 wt % at pH 10.

Overall, the DLS results suggest that H-bonding with small particle PNaSS end groups does not play a significant role. Instead, the shift in the location of the maximum points to changes in the self-assembly of HEUR itself. One possible candidate is pH-dependent solubility of the PEG backbone due to the formation of cationic suprapolyelectrolyte complexes at low pH.^49^ For example, the increased solubility of the PEG backbone at pH 4 could push the critical network formation to higher concentrations, while the effective positive charge could promote higher adsorbed amounts. Together these data also support the hypothesis from the adhesion data that stronger HEUR–particle interactions lead to HEUR enrichment at the surface of the film at low pH.

Zeta potentials in pH-adjusted solutions all showed a reduced magnitude relative to the unadjusted case due to the increased ionic strength, reducing the electric double layer thickness (Figure S11). This would allow more effective screening by the adsorbed polymer. There is a small trend for more effective screening in pH 4 solutions relative to the equivalent pH 10 conditions. However, the relative conductivities of hydronium and hydroxyl ions likely impact this result. Note that pH also has minimal influence on the rheology of the 10 wt % latex dispersions.

**Figure 8:**
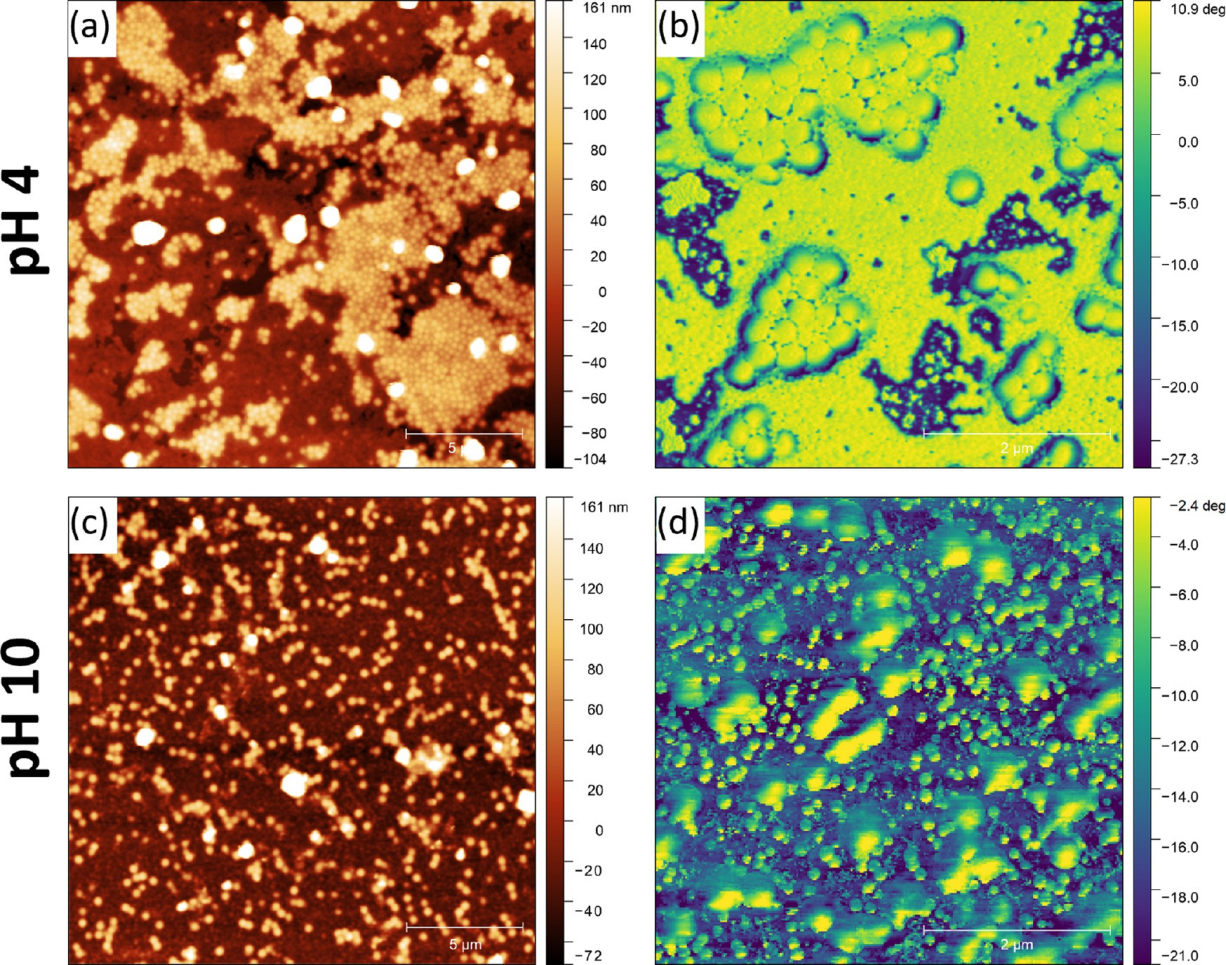
20 × 20 μm^2^ AFM topography images of films formed by diluting a dispersion containing a 10 wt % latex blend and 0.1 wt % HEUR10kC12 by a factor of 100 at (a) pH 4 and (c) pH 10. Corresponding 5 × 5 μm^2^ phase images at (b) pH 4 and (d) pH 10.

The AFM and light scattering data suggest that HEUR–latex interactions are crucial to driving stratification. However, these techniques cannot paint the full picture as AFM only probes the top layer of the film, and light scattering is measured at compositions far from those found during drying. Ideally, the cross section of the ∼30 μm film would be studied with Raman depth profiling^50^ or scanning electron microscopy. However, the chemical similarity of the latexes and the high rate of beam damage prevent the use of these techniques in our systems. An alternate approach to understanding the stratification is to reduce the thickness of the film, such that a monolayer may form. This is achieved by diluting the formulated dispersion by a factor of 100 and reducing the casting volume to 20 μL, as shown in Figure 8 for 0.1 wt % HEUR. Large differences in height make individual particles difficult to resolve in the topography images in Figure 8a,c. However, improved contrast is available in the phase images in Figure 8b,d. The phase images are qualitatively related to the mechanical properties of the sample, allowing areas of polymer and bare substrate to be reliably distinguished.

Similar to the full film AFM, the structure of the resulting monolayers is pH-dependent. At pH 4, the latex particles form aggregated patches consisting of either mixed large-small contacts, or solely small–small contacts, that bear a remarkable resemblance to the full film structures at 0.1 and 0.01 wt % HEUR (Figure 7a,b). The presence of both types of aggregates in the monolayer suggests changes in the equilibrium HEUR–latex interactions are insufficient to account for differences between the full film structures at 0.1 and 0.01 wt % HEUR.

In contrast, no significant patch formation is observed at pH 10. While glass–particle interactions are more significant for the low volume films, the glass slide at pH 4 still contains a significant negative charge with an expected zeta potential larger than −40 mV at low ionic strength.^51^ Therefore, the reduced patch formation at pH 10 suggests weaker HEUR-induced aggregation.

No significant pH effects occur in diluted structures at 0.01 and 1 wt % HEUR10kC12 as shown in Figure S12. At 0.01 wt % HEUR, well-dispersed particles comparable to pH 10 data in Figure 8 are present, again suggesting that the particles are stable against aggregation, while the structure at 1 wt % HEUR consists of microscale patches of mixed particles.

#### Mechanistic Insight into HEUR-Mediated Stratification

**Figure 9:**
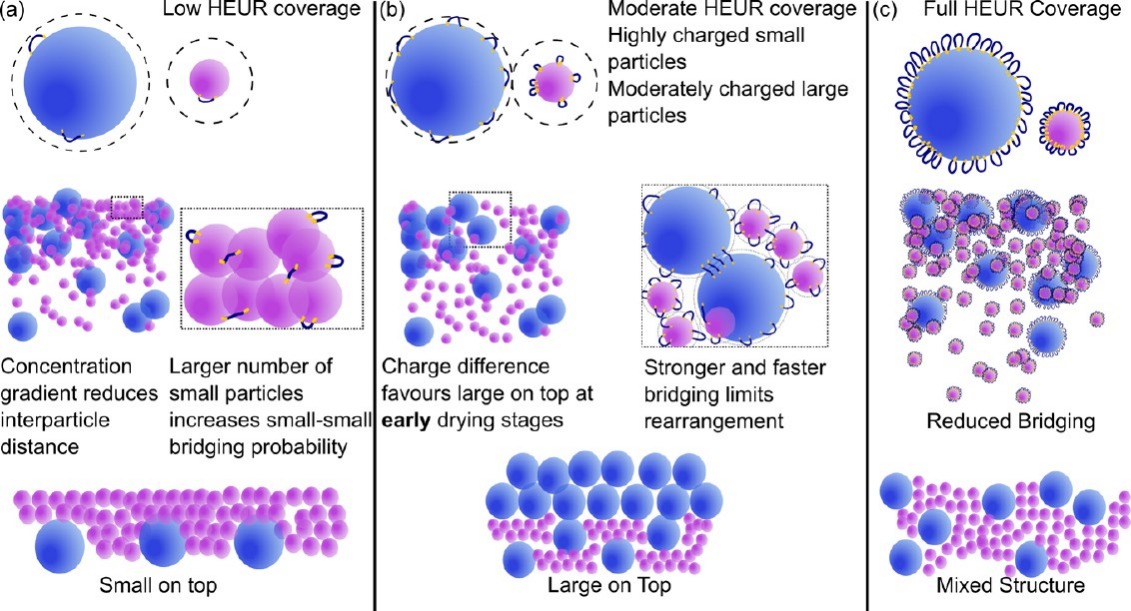
HEUR10kC12 concentration-dependent stratification mechanisms. (a) At 0.01 wt % low HEUR10kC12 coverage and a large:small number ratio of 1:9.5 favors small-on-top stratification. (b) At 0.1 wt % HEUR10kC12 large-on-top stratification is favored in early stages and then locked in. (C) At 1 wt % HEUR10kC12 the surfaces of particles are saturated leading to reduced bridging and a mixed structure similar to the HEUR10KC12-free case.

Measuring stratification of the HEUR–latex dispersions in situ is an open challenge.^8^ However, combining the observations allows the mechanisms outlined in Figure 9 to be proposed. Starting with the monolayer data in Figure 8, the results demonstrate that the structures at pH 4 and unadjusted conditions are aggregation driven. However, the long-term stability of the 10 wt % latex dispersions at HEUR concentrations up to 0.1 wt % and the stability of solutions in DLS measurements suggest that significant aggregation does not occur until the drying stage. In combination with the weak relationship between pH and the rheology, these data show the aggregation occurs at the high local latex concentration that develops during drying.

The aggregation should result from polymer bridging instead of depletion, as HEUR10kC12 strongly associates with the latex particles. While DLS shows particle identity-dependent adsorbed layer thicknesses at high HEUR10kC12 concentrations, there is no evidence for differences in adsorption at the low surface excesses relevant during drying. Furthermore, the diluted drying experiments in Figure 8 show that both small–small and mixed large–small aggregates can occur in the same dried sample at pH 4. This suggests that preferential interactions alone are insufficient to explain the structure.

The surface areas of each particle type in the dispersions used are approximately equal. Therefore, in the absence of preferential interactions, we assume that HEUR10kC12 is evenly distributed on each particle. Using values of the critical surface excess from the literature,^45^ we can estimate that at 0.01 wt % HEUR10kC12 the system is around 2.5% of the critical surface coverage (Table S2). This leads to a low probability of any single collision resulting in bridging (Figure 9a). In this case, the high number ratio of small to large particles of ∼9.5:1 favors a small-on-top structure because rate of aggregate growth is proportional to the number of particles.^52^

At 0.1 wt % HEUR10kC12, the system is around 25% of the critical surface coverage (Figure 9b). This would be expected to increase the probability of bridging of interparticle collisions, which would again favor small-on-top stratification rather than the large-on-top stratification that occurs experimentally. Selective sedimentation of the small particles may account for this structure. However, a more plausible explanation for the enrichment of large particles at the top of the film is the reduced magnitude of the zeta potential of large particles relative to that of small particles. The larger zeta potential and the corresponding mutual repulsion of the small particles lead to stronger collective diffusion away from the accumulation front and a relative enrichment of large particles at the air/water interface at the early stages of drying.^30^ Stickier collisions would allow the enrichment to be locked in before the collective diffusion effect disappears at higher particle concentrations. Stronger attraction between large particles due to the reduced curvature would enhance this effect.^53^

At 1 wt % HEUR10kC12 the surface of both particles is expected to be saturated, which greatly reduces the presence of direct bridging (Figure 9c). Here, the lack of significant aggregation leads to an observed uniform distribution of particles. Reduced aggregation is also sufficient to explain the structures occurring at pH 10. This reduced aggregation is also apparent in the diluted films in Figure 8. We speculate that weaker HEUR–latex interactions lead to reduced bridging at pH 10. The lower maximum adsorbed layer thickness of HEUR10kC12 and the shift of the maxima to lower HEUR10kC12 concentrations in Figure 6 as well as weaker adhesion in Figure S9 provide preliminary evidence for this.

## Conclusion

Self-stratification of colloidal dispersions during drying is an attractive method to form multilayer coatings in a single pass. In model systems stratification has been achieved through several approaches such as diffusiophoresis, controlled aggregation, and harnessing differences in surface chemistry.^3^ However, in practice, colloidal formulations are complex fluids where the influence of each component on others is difficult to predict. Rheology modifiers are particularly important components in coatings as the viscosity affects all stages of film formation. Therefore, in this work, we focused on understanding the influence of various rheology modifiers on self-stratification, necessary for further development of functional coatings.

We studied 10 wt % dispersions containing a 7:3 blend of large:small latex particles stabilized by NaSS units at their surface. In the absence of a rheology modifier, the particles do not stratify. Addition of between 0.004 and 1 wt % xanthan gum to this dispersion allowed the low shear viscosity and corresponding Pe to be modulated by several orders of magnitude. Importantly, the rheology of the system is largely unaffected by the presence of latex particles, which suggests minimal latex–xanthan gum interactions. Subsequently, the degree of stratification can be controlled with no stratification at Pe_S_ extremes and small-on-top stratification achieved at intermediate Pe_S_, despite the relatively low size ratio of the particles. These results compare favorably with the jamming front theory of diffusiophoresis-based stratification.^19^

The addition of between 0.01 and 1 wt % Carbopol 940 to the latex dispersion has a similarly large influence on the viscosity as xanthan gum. Unexpectedly, the large changes in viscosity had a minimal effect on stratification. Instead, the resulting structure is largely determined by Carbopol 940–latex interactions. Here, at pH 3 strong rheology modifier–particle interactions result in laterally segregated structures at Carbopol 940 concentrations of 0.1 wt % and above. In contrast, weaker interactions at pH 8 led to a minor enhancement of large particles at the top of the film.

The associative rheology modifier HEUR10kC12 is also found to have strong interactions with the studied latexes. Compared to the other rheology modifiers, the change in viscosity is minor, which allows the influence of HEUR10kC12–latex interactions to be isolated from viscosity effects. We found a nonmonotonic relationship between HEUR10kC12 concentration and stratification with small-on-top, large-enriched, and randomly distributed structures occurring at 0.01, 0.1, and 1 wt % HEUR10kC12, respectively. Polymer bridging kinetics is found to drive the concentration dependent stratification. Significantly, the reduction of interparticle distance at the drying front allows the aggregation driven stratification of dispersions that are colloidally stable prior to application. Reducing the strength of the HEUR10kC12–latex interactions by raising the pH led to a reduced stratification.

In conclusion, in the absence of rheology modifier–latex interactions, stratification can be controlled and even predicted via modifying the Pe_S_. However, the presence of interactions with rheology modifiers can override the influence of Pe_S_ and result in a rich array of surface structures. Our work advances this understanding and provides valuable insights that can be harnessed when making a choice of rheology modifiers for advanced self-stratifying coatings.

## Supporting Information Available

Viscosity vs shear rate curves for HEUR systems (Figure S1); viscosity vs HEUR concentration curves (Figure S2); interparticle distance calculations; Carbopol films topography (Figure S3) and adhesion maps (Figure S4) as a function of pH; DLS and zeta supplementary calculations; intensity vs hydrodynamic radius curves for large (Figure S6) and small (Figure S7) particles in the presence of HEUR; zeta potential curves as a function of HEUR concentration (Figure S8); adhesion maps and histograms for 1 wt % HEUR films (Figure S9); AFM force curves for a 1 wt % HEUR film (Figure S10); zeta potential in pH-controlled solutions (Figure S11); AFM topography maps of diluted dispersions (Figure S12) (PDF)
