# Supplementary material for: One Step Closer to Coatings Applications Utilizing Self-Stratification: Effect of Rheology Modifiers
Source: ACS Appl Polym Mater. 2023 Jul 31;5(8):6672–84. doi: 10.1021/acsapm.3c01288 (PMC10425952; doi:10.1021/acsapm.3c01288)
Supplement: Supplementary file 1 — ap3c01288_si_001.pdf [file ap3c01288_si_001.pdf]

# Supporting Information: One step closer to coatings applications utilizing self-stratification: Effect of rheology modifiers

*Timothy J. Murdoch,[†] Baptiste Quienne,[‡] Maialen Argaiz,[§] Radmila Tomovska,[§] Edgar Espinosa,[#] Franck D'Agosto,[#] Muriel Lansalot,[#] Julien Pinaud,[‡] Sylvain Caillol[‡] and Ignacio Martín-Fabiani[†,*]*

[†]Department of Materials, Loughborough University, LE11 1RJ Loughborough, United Kingdom

[‡]ICGM, Univ Montpellier, CNRS, ENSCM, 34293 Montpellier Cedex 5, France.

[§]POLYMAT and Departmento de Química Aplicada, Facultad de Ciencias Químicas, University of the Basque Country, UPV/EHU, Joxe Mari Korta Zentroa, Tolosa Hiribidea 72, Donostia-San Sebastian, 20018, Spain

[#]Univ Lyon, Université Claude Bernard Lyon 1, CPE Lyon, CNRS, UMR 5128, Catalysis, Polymerization, Processes and Materials (CP2M), 43 Bd du 11 novembre 1918, 69616 Villeurbanne, France

*corresponding author: Ignacio Martín-Fabiani i.martin-fabiani@lboro.ac.uk

## Rheology Associated Content

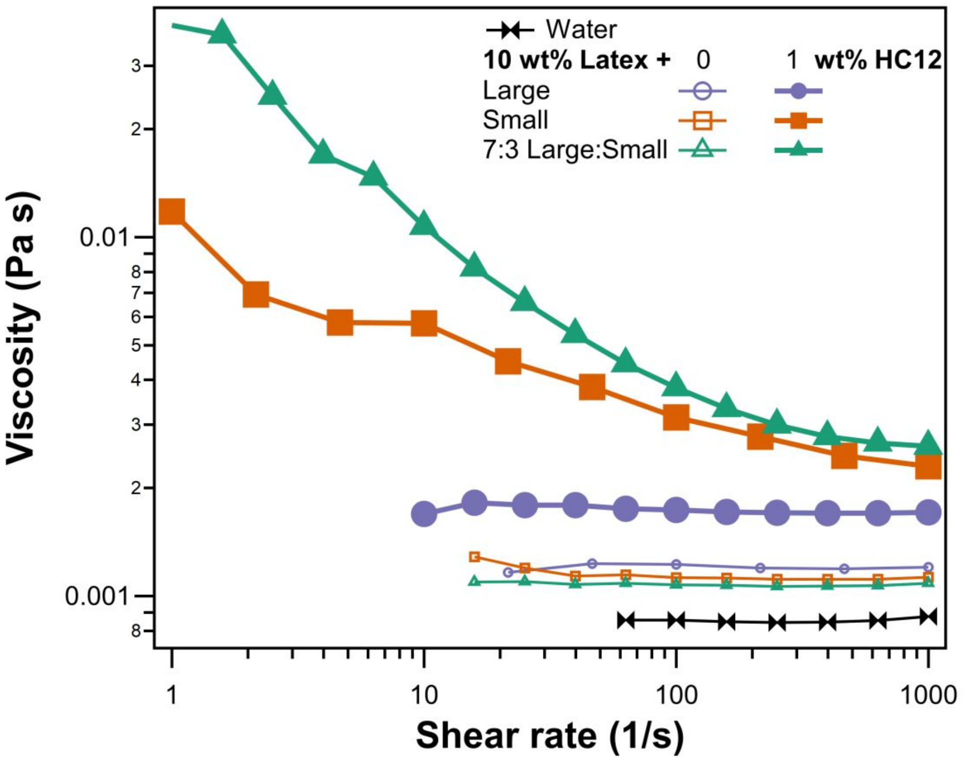


**Figure S1 –** Viscosity vs shear rate for 10 wt% latex blends with and without 1 wt% HEUR10kC12

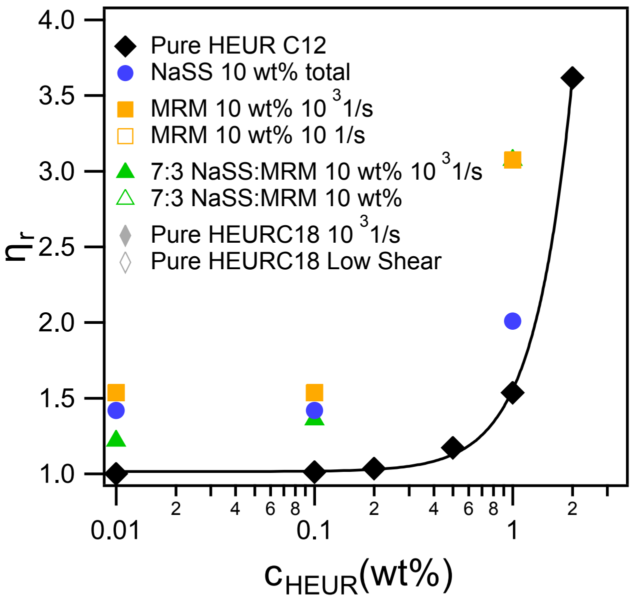


**Figure S2 -** Relative viscosity vs HEUR10kC12 concentration for solutions with and without 10 wt% latex particles.

The average interparticle distance ($h$) of a dispersion of spherical particles of radius $a$ with volume fraction ($\phi$) is given by:

$$h = 2a\left[\left(\frac{\pi}{2\sqrt{6}\phi}\right)^{\frac{1}{3}} - 1\right] \quad \text{(S1)}$$

Representative values of $h$ for the particles in this study are given in Table S1.

**Table S1 –** Representative interparticle distance for different volume fractions of the particles used in this study. $\phi$ for the solutions prior to casting is ~0.1.

| ***R*** **(nm)** | 48 | 135 |
|---|---|---|
| $\phi$ | ***h*** **(nm)** | ***h*** **(nm)** |
| 0.1 | 82 | 231 |
| 0.2 | 46 | 128 |
| 0.3 | 28 | 77 |
| 0.4 | 16 | 46 |
| 0.5 | 8.3 | 23 |
| 0.6 | 2.2 | 6.1 |

# Carbopol940 Containing Films Associated Content

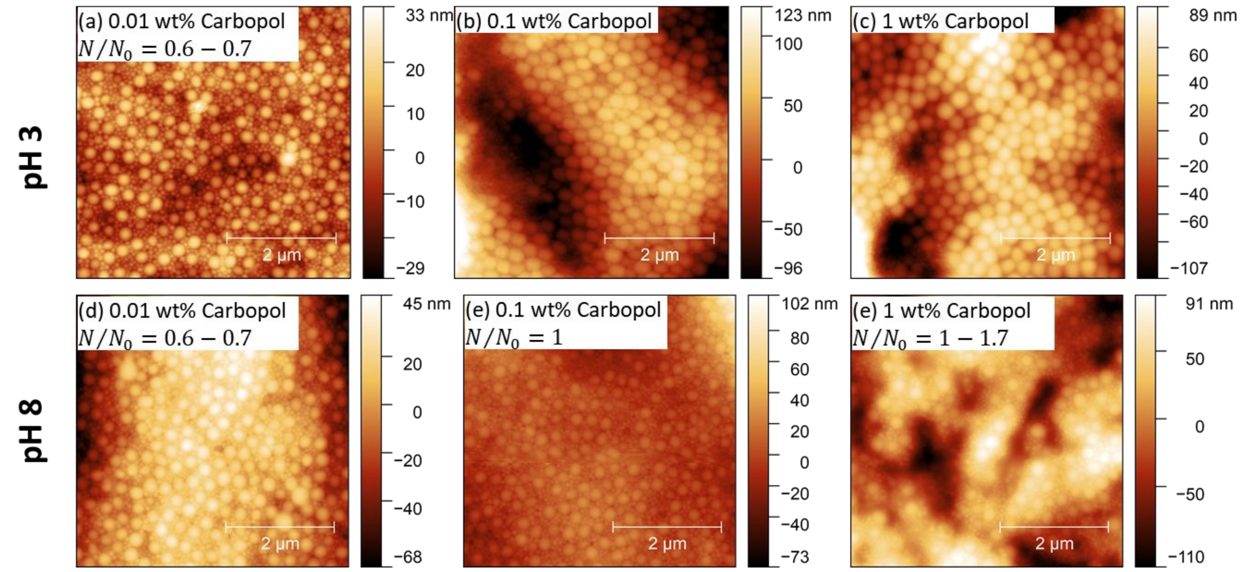


**Figure S3 -** 5 × 5 µm AFM topography images of films formed from 10 wt% latex blend with varying concentrations of Carbopol940 at either pH 3 or 8

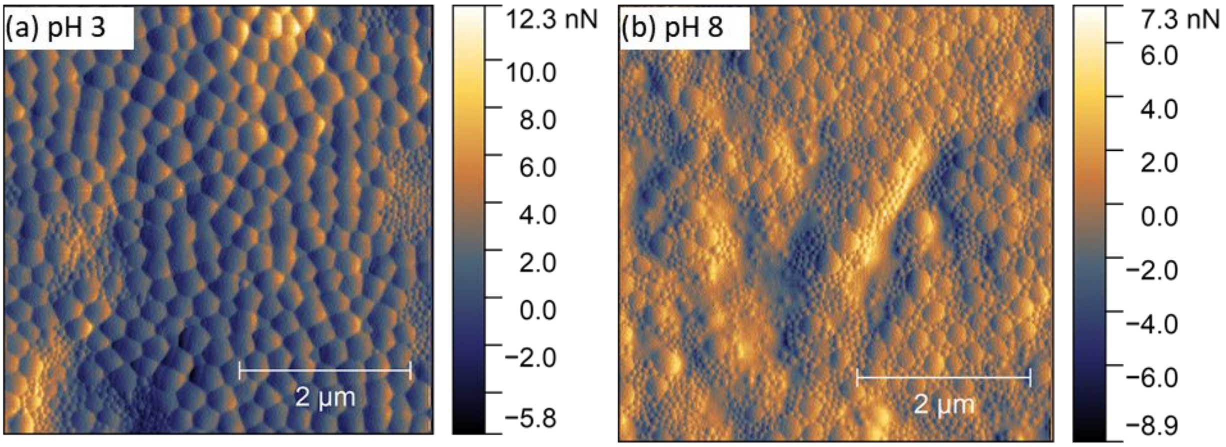


**Figure S4** 5 × 5 µm AFM adhesion maps of films formed from 10 wt% latex blend with 1 wt% Carbopol940 at either (a) pH 3 or (b) pH 8

# HEUR10kC12 DLS and Zeta Potential Associated Content

Following the approach of Pham et al.,[1] the Alexander-de Gennes scaling theory for dense brushes in a good solvent predicts the brush thickness, $\delta$, on a spherical particle radius *a* >> $\delta$ to be:

$$\delta \sim L\left(\frac{\sigma v}{6l}\right)^{1/3} = L\left(\frac{l^2 \sigma v}{6\ l^3}\right)^{1/3} \qquad \text{(S2)}$$

where $L$ is the contour length, $\sigma$ is the grafting density, *l* is the Kuhn length, and $v$ is the excluded volume parameter. Fits to osmometry data in the literature yield $\frac{v}{l^3} = 0.23$.[2] Simulations give a critical

value of $\sigma_c \sim 0.08 \frac{\text{chains}}{\text{nm}^2}$ for direct adsorption of HEUR with C12 hydrophobes of similar molar mass $M_r$.[3] This value is comparable to typical experimental values of $\sigma$ for higher $M_r$ HEUR.[4] $L$ is calculated to be 63.6 nm using a monomer length of 0.28 nm.[5] In the case of both a hair-pin conformation with both hydrophobic groups adsorbed we use effective grafting density and contour length values of $\sigma_{\text{eff}} = 2\sigma$ and $L_{\text{eff}} = \frac{L}{2}$.

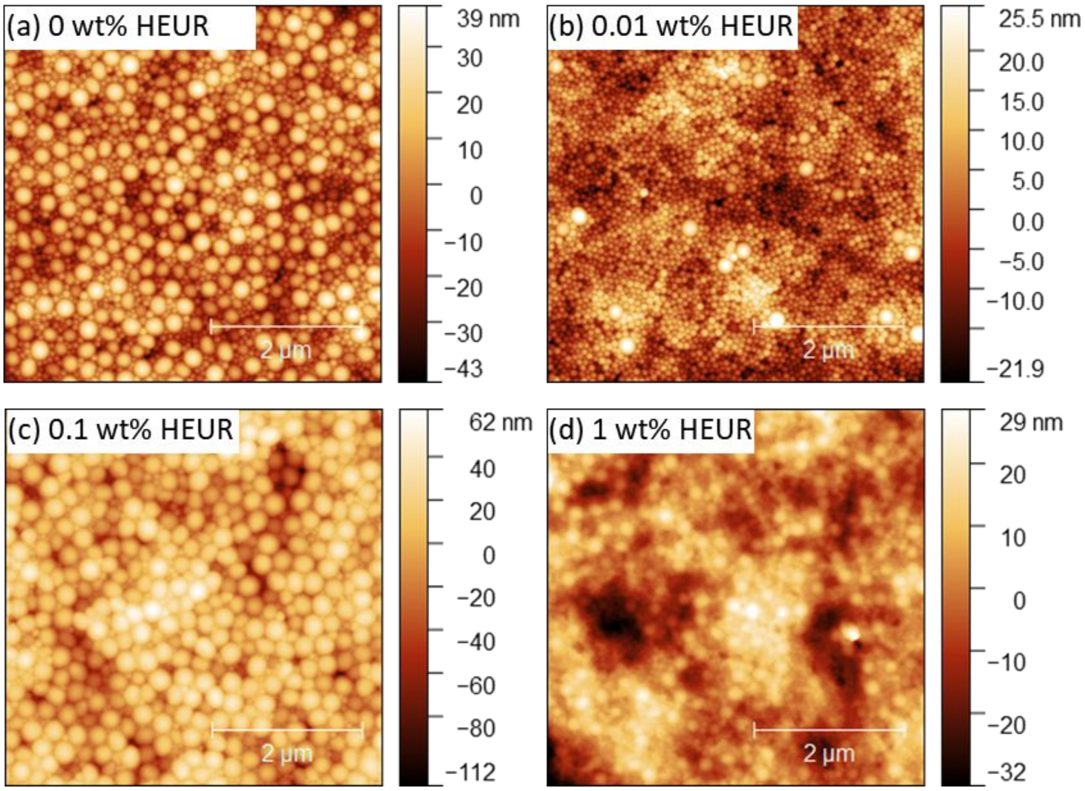


**Figure S5 -** 5 × 5 µm AFM topography images of films formed from 10 wt% latex blend with a volume fraction ratio of 85:15 large:small particles with varying concentrations of HEUR10kC12

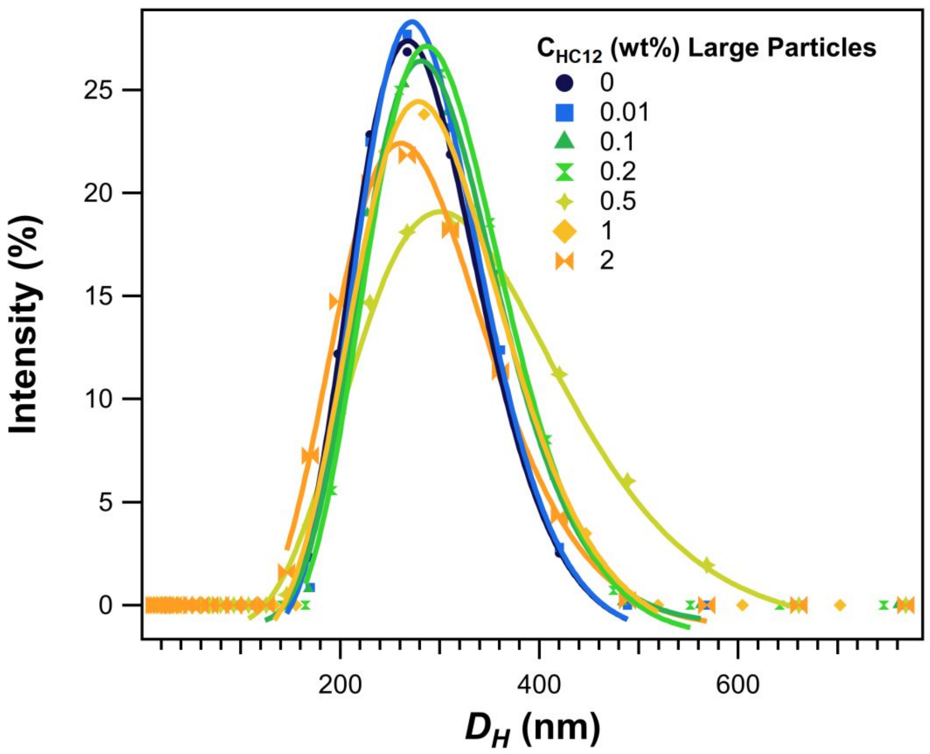


**Figure S6 –** Intensity vs hydrodynamic radius of large particles as a function of HEUR10KC12 concentration

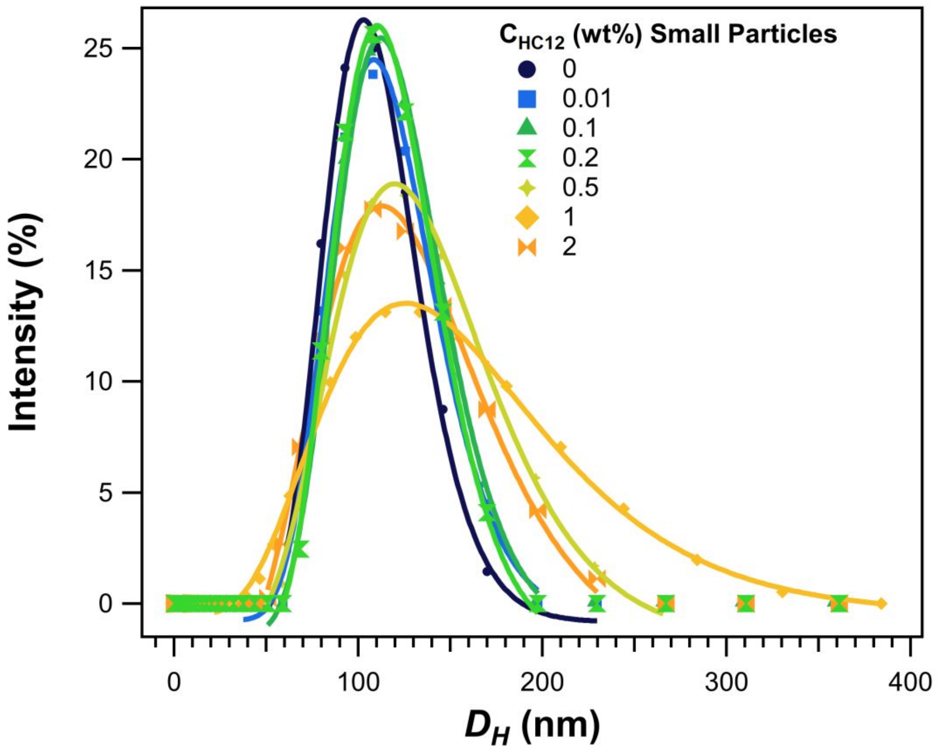


**Figure S7 –** Intensity vs hydrodynamic radius of small particles as a function of HEUR10KC12 concentration

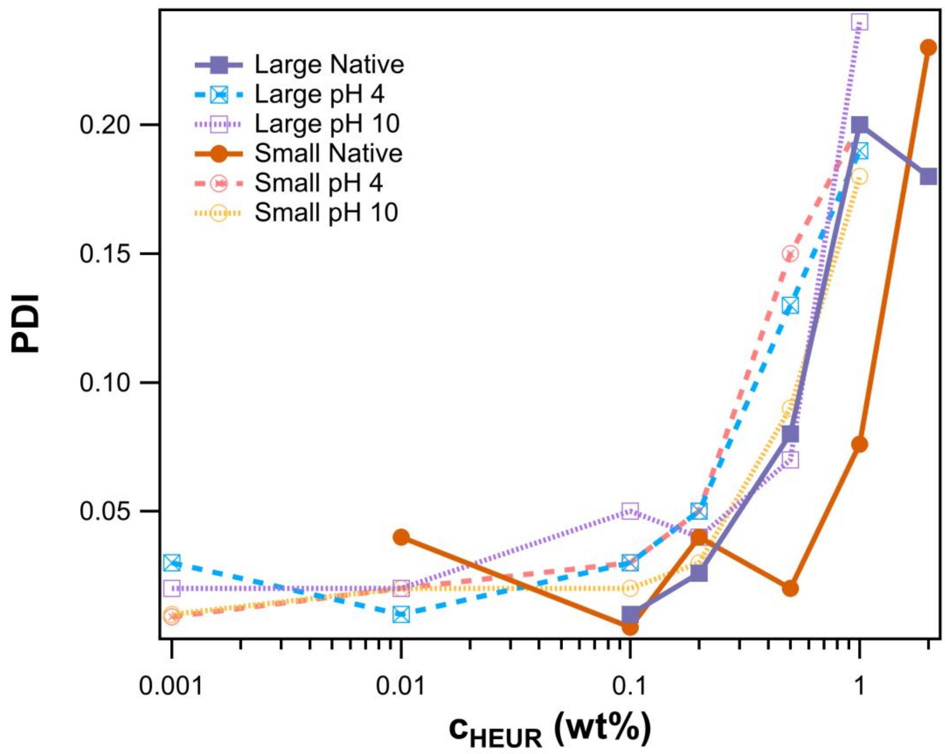


**Figure S8 –** PDI vs HEUR10kC12 concentration for large and small latexes

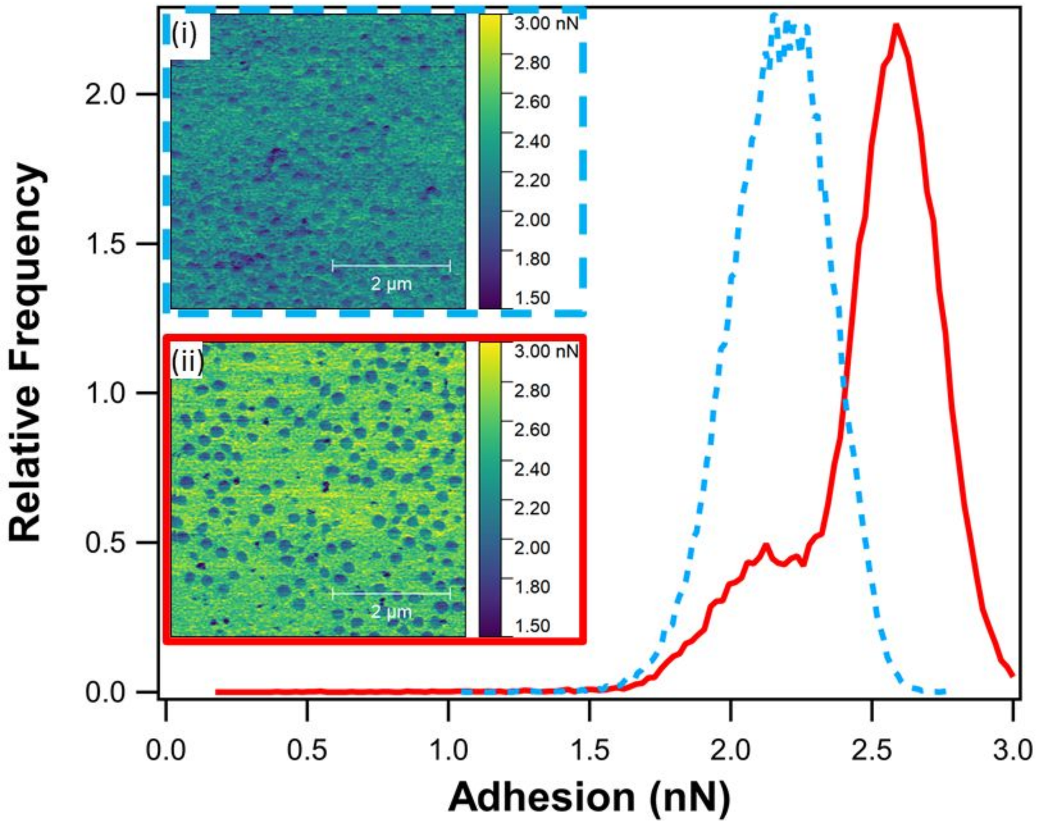


**Figure S9 -** Adhesion histograms of 10 wt% latex blend in 1 wt% HEUR10kC12 at pH 10 (blue dashes) and 4 (solid red). Inset: Adhesion maps at (i) pH 10 and (ii) pH 4 used to generate the histograms.

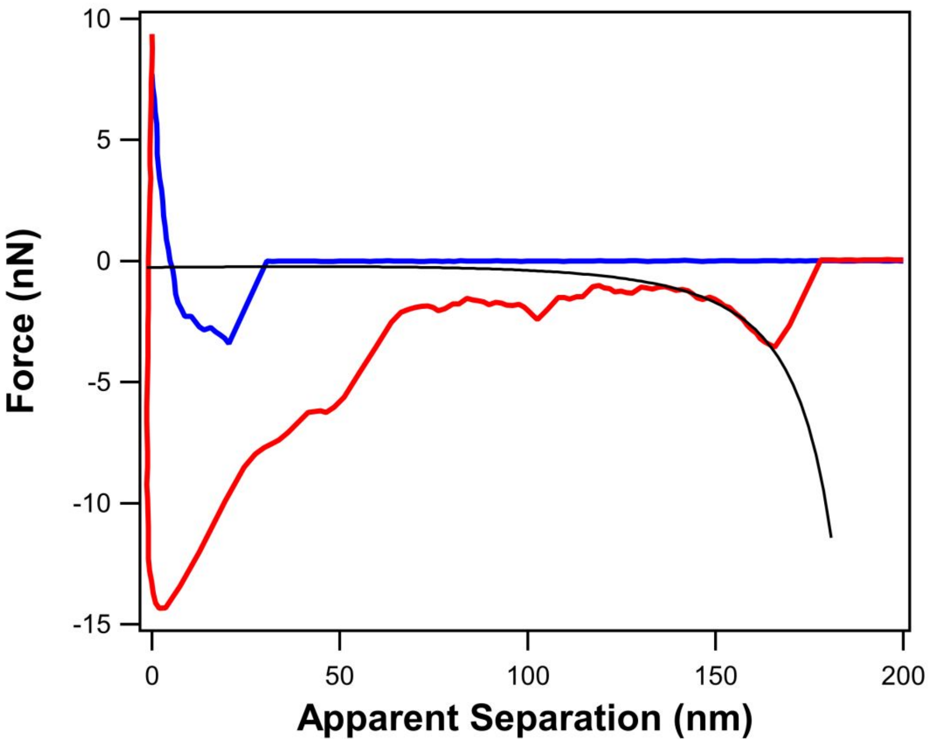


**Figure S10 –** Force vs apparent separation of an AFM tip interacting with the surface of a film formed from a dispersion containing 10 wt% latex mixture and 1 wt% HEUR10kC12. The blue line corresponds to the tip approaching the surface, while the red line corresponds to retraction. At small separations many polymer chains are likely in contact with the tip leading to large adhesion. As the tip retracts chains stretch and detach continuously leading to a smooth reduction in the magnitude of adhesion. At large distances there is a higher chance that a single chain is being stretched, as shown by fitting a worm-like chain model to the final pull off event prior to returning to baseline.

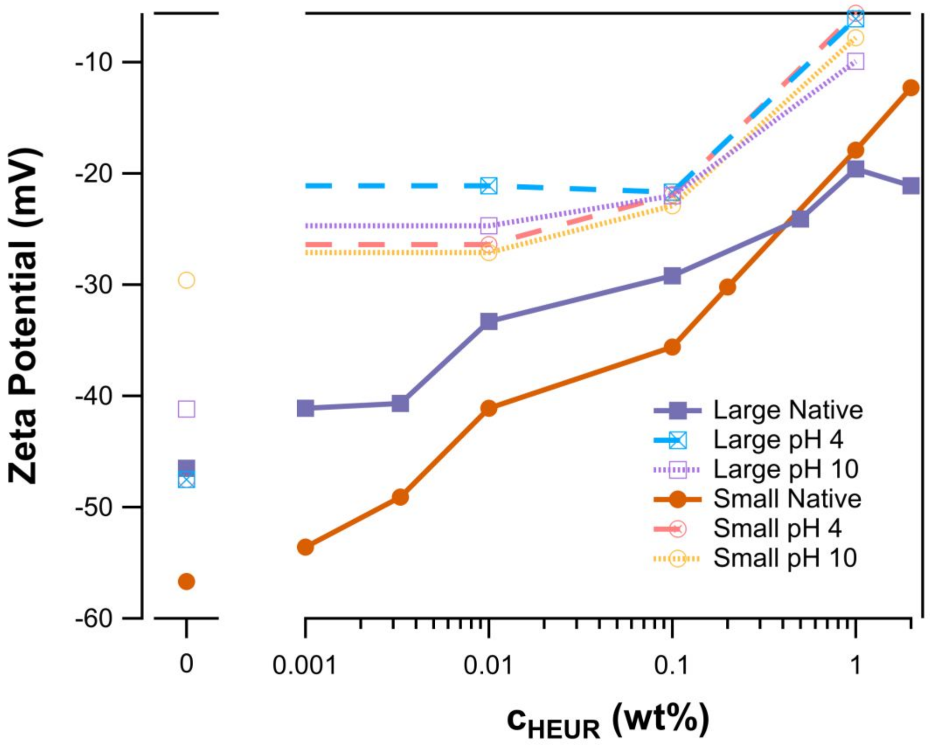


**Figure S11 –** Zeta potential in pH controlled solutions

Assuming all HEUR molecules are attached to the latex surface the effective HEUR grafting density is given by:

$$\sigma = \frac{\frac{n_{\text{chains}}}{V}}{\frac{SA_{\text{total}}}{V}} = \frac{\frac{c\rho N_{\text{A}}}{M}}{\frac{3\phi}{a}} = \frac{c\rho N_{\text{A}}}{M}\frac{a}{3\phi} \qquad \text{(S3)}$$

where $\frac{n_{\text{chains}}}{V}$ is the number of HEUR chains per volume of solution, $\frac{SA_{\text{total}}}{V}$ is the total latex surface area per volume of solution, $c$ (g polymer/g solution) is the mass concentration of HEUR in solution, $\rho$ is the solution mass density, $N_{\text{A}}$ is Avogadro's number, $\phi$ is the volume fraction of latex particles in solution, and $M$ is the HEUR molecular weight. Note an alternate representation of S3 is adapted from Larson et al. where the HEUR concentration prior to mixing was used.[4] In the case of binary particle blend with relative fraction of small and large particle $x_{\text{s}}$ and $x_{\text{l}}$:

$$\sigma = \frac{c\rho N_A}{M}\frac{a_s a_l}{3\phi(a_s x_l + a_l x_s)} \qquad \text{(S4)}$$

Course-grained molecular dynamics simulations of molecules comparable to HEUR10kC12 suggest a critical surface coverage, where surfaces are more or less completely covered by HEUR, of around 0.08 chains nm$^{-2}$. Using this value, we can estimate the % critical surface coverage using Equation S4. Results for different blends are given in Table S2.

**Table S2 -** % of Critical HEUR10kC12 surface coverage as a function of volume fraction of a 7:3 mixture of particles with $a_l$ = 135 and $a_s$ = 48

| $C_{HEUR}$ (wt%) | $C_{latex}$ (wt%) | $\phi$ | % Surface Coverage |
|---|---|---|---|
| 0.01 | 10 | 0.09 | 2.5 |
| 0.1 | 10 | 0.09 | 24.5 |
| 1 | 10 | 0.09 | 245 |
| 0.001 | 0.05 | 0.0004 | 50 |
| 0.01 | 0.05 | 0.0004 | 500 |

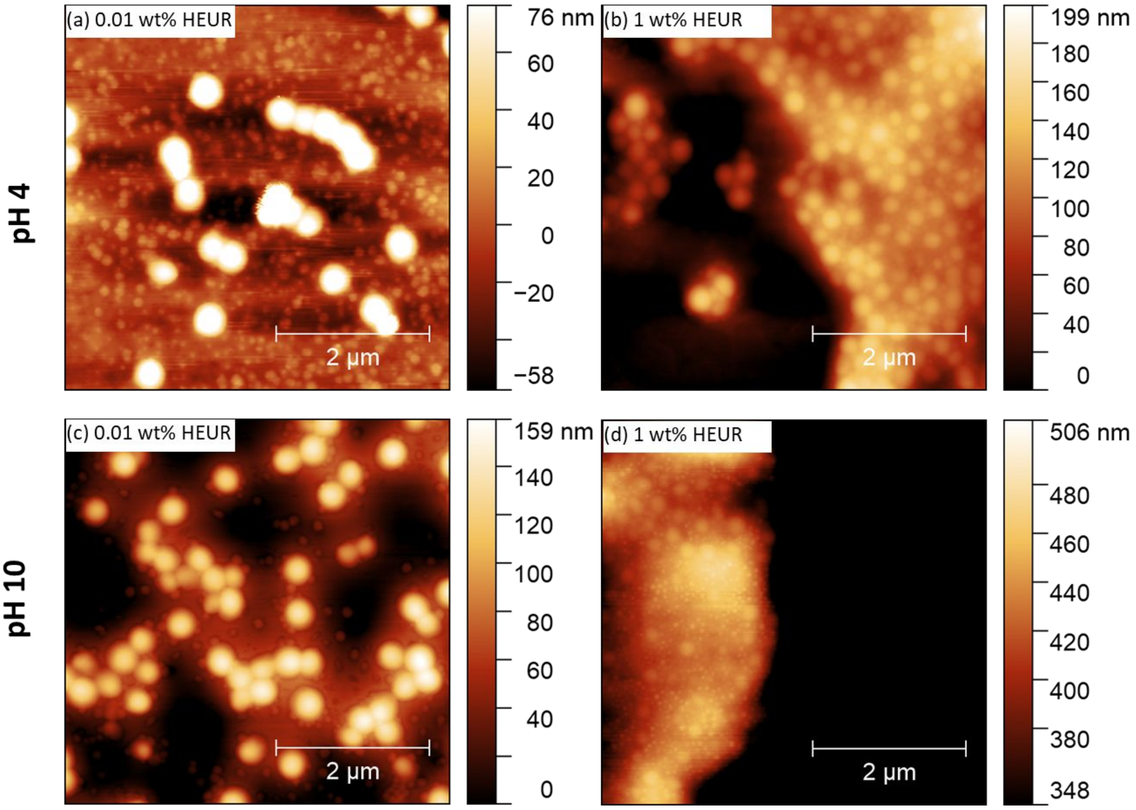


**Figure S12** 5 × 5 μm AFM topography images of latex and HUER10kC12 dispersions diluted by a factor of 100 prior to casting. Concentrations in labels refer to pre-dilution conditions. At 0.01 wt% HEUR10kC12 (a,c) small particles are well dispersed while large particles have mixture of small clusters and well dispersed particles. At 1 wt% large patches of particles with a similar composition to the dispersion prior to casting are observed.

# References

(1) Pham, Q. T.; Russel, W. B.; Lau, W. The Effects of Adsorbed Layers and Solution Polymer on the Viscosity of Dispersions Containing Associative Polymers. *J. Rheol. (N. Y. N. Y).* **1998**, *42* (1), 159–176. https://doi.org/10.1122/1.550934.

(2) Zhang, W.; Travitz, A.; Larson, R. G. Modeling Intercolloidal Interactions Induced by Adsorption of Mobile Telechelic Polymers onto Particle Surfaces. *Macromolecules* **2019**, *52* (14), 5357–5365. https://doi.org/10.1021/acs.macromol.9b00775.

(3) Ginzburg, V. V; Van Dyk, A. K.; Chatterjee, T.; Nakatani, A. I.; Wang, S.; Larson, R. G. Modeling the Adsorption of Rheology Modifiers onto Latex Particles Using Coarse-Grained Molecular Dynamics (CG-MD) and Self-Consistent Field Theory (SCFT). *Macromolecules* **2015**, *48* (21),

8045–8054. https://doi.org/10.1021/acs.macromol.5b02080.

(4) Larson, R. G.; Van Dyk, A. K.; Chatterjee, T.; Ginzburg, V. V. Associative Thickeners for Waterborne Paints: Structure, Characterization, Rheology, and Modeling. *Prog. Polym. Sci.* **2022**, *129*, 101546. https://doi.org/10.1016/j.progpolymsci.2022.101546.

(5) Osterhelt, F.; Rief, M.; Gaub, H. E. Single Molecule Force Spectroscopy by AFM Indicates Helical Structure of Poly(Ethylene Glycol) in Water. *New J. Phys.* **1999**, *1*, 6.1-6.11.
